# Covalent, Non-Covalent, Encapsulated Nanodrug Regulate the Fate of Intra- and Extracellular Trafficking: Impact on Cancer and Normal Cells

**DOI:** 10.1038/s41598-017-06796-7

**Published:** 2017-07-25

**Authors:** Sang-Woo Kim, Yeon Kyung Lee, Sang-Hyun Kim, Jun-Young Park, Dong Un Lee, Jungil Choi, Jeong Hee Hong, Sanghyo Kim, Dongwoo Khang

**Affiliations:** 1 Lee Gil Ya Cancer and Diabetes Institute, Gachon University, Incheon, 21999 South Korea; 2 Department of Pharmacology, School of Medicine, Kyungpook National University, Daegu, 41566 South Korea; 3 Gyeongnam Department of Environment Toxicology and Chemistry, Korea Institutes of Toxicology, Jinju, 52834 South Korea; 4 Department of Physiology, College of Medicine, Gachon University, Incheon, 21999 South Korea; 5 Department of Bionanotechnology, Gachon University, Seongnam, 13120 South Korea

## Abstract

Drugs need to be designed to access the designated intracellular organelle compartments in order to maximize anticancer efficacy. This study identified that covalently conjugated, non-covalent polyethylene glycol coated and encapsulated nanodrugs selectively influence drug uptake, the intracellular and extracellular trafficking of cancer cells. The types of nano conjugation modulated intracellular dynamics associated with differential impact on anti-cancer efficacy, but also induced differential cytotoxicity on cancer versus normal cells. In conclusion, this study demonstrated the importance of selecting the appropriate type of nano-conjugation for delivering organelle specific, active chemotherapeutic agents through controlled intracellular trafficking.

## Introduction

The global gross market value of targeted chemotherapies has already surpassed that of non-targeted therapeutics a decade ago^1, 2^. As a next consideration, the optimal nanodrug delivery to specific intracellular cancer organelles gains critical importance. This is because the next generation of anti-cancer nanodrugs should not only feature increased cell targeting efficacy, but should also implement designs that transport the drug to the designated intracellular organelle with minimal drug efflux.

It is well known that uptake of nanodrugs in cancer cells involves adenosine triphosphate (ATP)-dependent uptake mechanisms (i.e., dynamin-dependent), such as macropinocytosis, clathrin-coated pits, caveolae and associated intracellular trafficking of drugs in cancer cells were significantly different from the trends observed in normal cells^3–6^. Specifically, cancer cells typically exhibit highly upregulated amounts of membrane receptors, compared to normal cells, those membrane receptors mediate endocytosis, as they are necessary to maintain cellular energy metabolism for cancer survival^3^. Therefore, internalized nanodrugs in cancer cells might experience more complex intracellular trafficking compared to that in normal cells. In addition, it has been well documented that cancer cells possess more endolysosomal compartments with acidic pH and abundant enzymes in the lysosomes (Lys), and exhibit higher redox potential (related to reduction/oxidation homeostasis) compared with normal cells^7, 8^. These differences manifest as varying levels of therapeutic nanodrug efficacy and can elicit selective toxicity to cancer and normal cells.

Intracellular drug delivery is also influenced by the physiochemical properties of nanomaterials (e.g., size, shape, charge and surface modification)^9, 10^. For example, materials featuring a nanoparticle size of approximately 60 nm were internalized through caveolin-dependent endocytosis and rapidly transported into the Golgi or nucleus^4, 11^. In contrast, nanomaterials, whose particle size below 120 nm, were internalized through clathrin-dependent endocytosis, transported to early endosomes (EE) and late endosomes (LE) and, ultimately, accumulated in the Lys^12, 13^. Particles whose size dimensions were at the micron-scale (i.e., 0.5~10 μm) were internalized through macropinosomes and fused with Lys^14^.

Surface functionalization and surface charge are both independent factors those mediate intracellular targeted delivery, particularly useful for targeting delivery to mitochondria^15, 16^. This is due to the highly negative membrane potential of the mitochondrial membrane (i.e., approximately −220 mV)^17^ which attracted positively-charged poly(lactide-co-glycolide) (PLGA) nanoparticles (+30 mV) that escaped from early endosomes, and concentrated in the mitochondria^18^. Surface attachment of target agents (e.g., peptides with cations) was also effective. For example, gold nanorods conjugated with cetyltrimethylammonium (CTAB), a cation, accumulated in the mitochondria of A549 lung cancer cells^5^. Mitochondria-targeting gold nanorods escaped into the cytosol from endosomes/lysosomes and abruptly changed the mitochondrial membrane potential by increasing cellular reactive oxygen species (ROS) levels, which, ultimately, culminated in cell death^5^.

To efficiently target specific subcellular organelles, the controlled release of conjugated or encapsulated drug at the specific cellular environments is desirable. Traditionally, nano-sized encapsulation (e.g., liposome, lipid or polymer-based nanoparticles, and nanoemulsions) was widely known form of anti-cancer nanodrug^19^. In addition, nano conjugation strategies include covalent (e.g., amide bonds, disulfide bonds, ester bonds, carbamate bonds, and radical coupling) and non-covalent conjugation (e.g., polyethylene glycol (PEG) coat by hydrophobic interaction and π-π stacking interaction)^20^.

Intracellular trafficking and endosomal escape of lipid-encapsulated nanodrugs were quantitatively investigated *in vitro* (i.e., HeLa cells), and *in vivo* (i.e., primary mouse liver cells models)^21^ and revealed extremely low siRNA release into the cytosol from liposomal encapsulation (i.e., only 1–2%). Similarly, PEG coated nanodrugs easily released into the cytosol and induced an unpredictable fate of intracellular trafficking^22^. Covalently-conjugated nanodrugs showed the drug release by cleaving conjugated bonds under internal or external stimuli (e.g., pH, enzyme, light, and thermal energy)^23, 24^. Higher degrees of covalent conjugation (i.e., conjugated with more doxorubicin molecules) also improved drug accumulation in the nucleus and exhibited increased drug retention time in HepG2 liver cancer cells^25^. To maximize anti-cancer efficacy, covalent conjugation has also been utilized for dual targeting schemes, comprising both a mitochondria-damaging drug (e.g., α-tocopheryl succinate) and nucleus-damaging drugs (e.g., cisplatin, doxorubicin and paclitaxel)^26^.

Different anti-cancer nanodrug efficacy on cancer versus normal cells and understating on associated intracellular, extracellular dynamics by various conjugation types of nanodrugs on both cancer and normal cells are still unclear^27^.

This study elucidated that the altered localization of doxorubicin (DOX) at the subcellular organelle level was influenced by different conjugation types of carbon nanotubes (CNT) and, furthermore, identified a difference in anticancer efficacy of carbon nanotube-doxorubicin by the additional conjugation of overexpressed cancer-specific receptor antibody without the elevation of cytotoxicity to normal cells.

## Results and Discussion

### Nanodrugs and materials characterization

**Figure 1:**
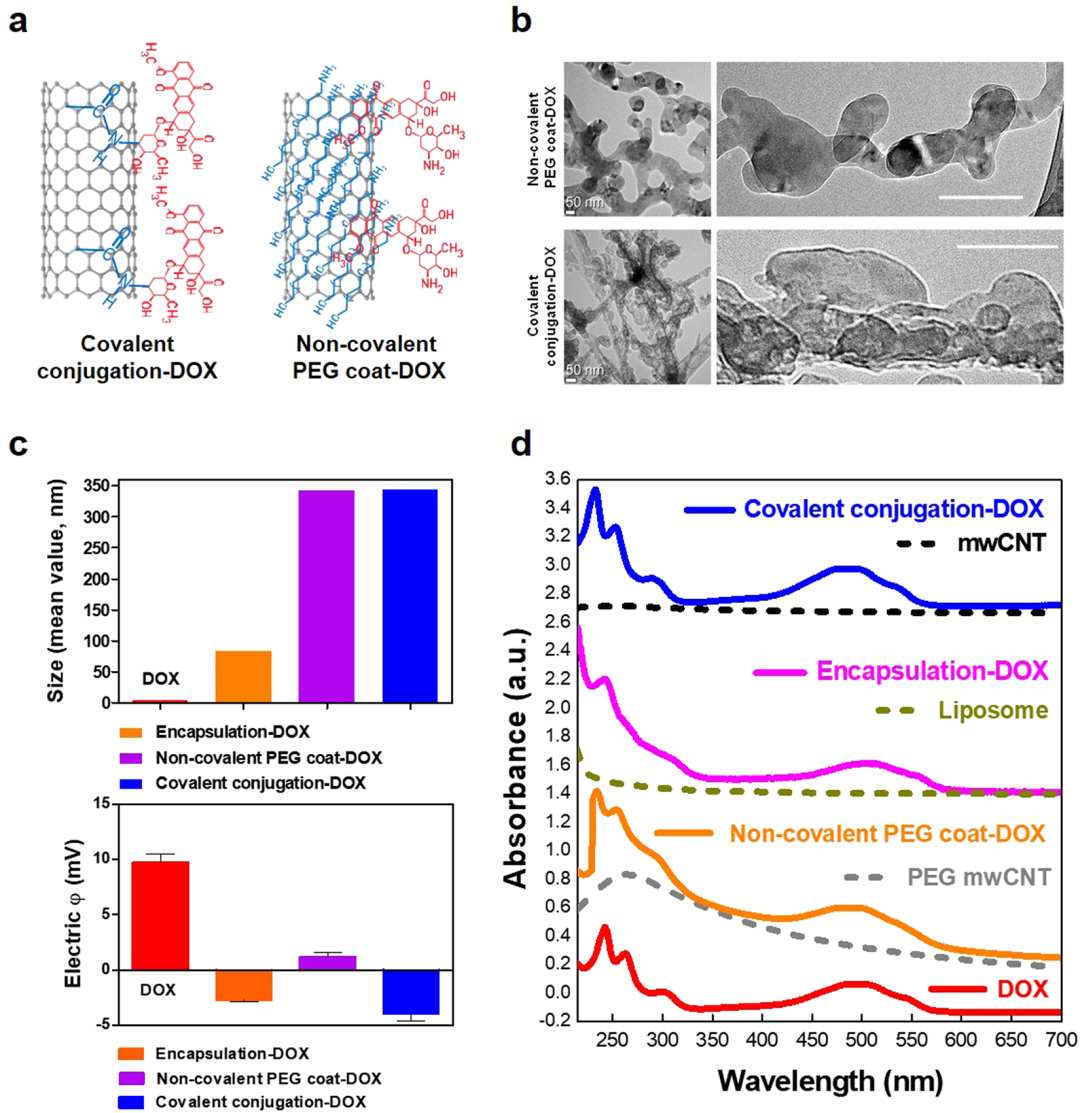
Physiochemical characteristics of nanodrugs (**a**) Schematic illustration of the various types of nanodrugs. (**b**) Cryo-TEM images (left: low resolution, right: high resolution) revealed different morphologies for the various types of nanodrugs investigated. Encapsulation-DOX exhibited a round shape. Images of the PEG coated drugs (i.e., DOX) showed that the drugs were embedded into the PEG coated CNT (see Figure S1b). Covalent conjugation-DOX showed direct exposure of the drug molecules on the covalently-linked CNT. Scale bar is 100 nm (right). (**c**) The particle sizes (upper) of the nano-conjugated drugs ranged from 80 nm to 350 nm and the zeta potential (bottom) showed negative electrical potentials in neutral pH buffer (i.e., PBS 7.2) with the exception of DOX. (**d**) The amount of attached DOX for each type of nano-conjugation was determined by UV-vis absorption. Results are depicted as follows: covalent conjugation (blue), liposomal encapsulation (pink), PEG coat (orange) and DOX (red). The base nanomaterials were used as a reference for calculating the amount of attached DOX.

Three different types of nanodrug were prepared to investigate their effect on intracellular dynamics. Specifically, encapsulation-DOX (DOXOVES^®^), PEG coat-DOX (i.e., using non-covalent interactions with CNT) and covalent conjugation-DOX (i.e., using amide bonds with CNT) were used (Fig. 1a and Figure S1a). The physical size of the tested nanodrugs ranged from 80–350 nm (Fig. 1c and Figure S1b). Whereas encapsulation-DOX was approximately 80 nm, the size of covalently conjugated and PEG coated nanodrugs were around 340–350 nm (Fig. 1c and Figure S1b). Note that the long cylindrical length of CNT should not be considered the effective (or practical) dimension during cellular uptake^28^ because the upright cylindrical direction of CNT during cellular uptake to minimize surface tension is thermodynamically favorable^29^. The unconjugated drugs in their original form are a few nanometers (DOX) in size (Fig. 1c and Figure S1b). The polydispersity index (PDI) was less than or equal to 0.5 for all tested samples (see Figure S2a) and the electrical potential of all tested drugs indicated negative charges in neutral buffers (i.e., pH of PBS = 7.2) except for DOX and PEG coat-DOX (Fig. 1c). The net negative charge of nanodrugs indicates no direct electrical attraction with mitochondria (by electric repulsion) and, thus, mitochondrial accumulation of nanodrugs would not be driven by attractive charge interactions with nanodrugs. Electrical potential changes of DOX depended on the pH environment (Figure S2b). In acidic buffer (pH = 4), the net potential of DOX slightly increased (but non-significantly compared to the neutral buffer with a pH = 7) (Figure S2b).

Cryo-transmission electron microscopy (Cryo-TEM) images showed different morphologies for each nanoconjugation type (Fig. 1b and Figure S1c). The covalently conjugated DOX showed direct exposure of the DOX molecules on CNTs, but PEG coated drug (DOX) showed drug molecules embedded into the PEG coated CNT (Fig. 1b). The embedded CNT in PEG coated polymers were confirmed by the presence of contrast regions identified by high resolution Cryo-TEM imaging (data not shown). Liposomal DOX showed round-shaped liposomes encapsulating DOX drugs (Figure S1c).

### Drug loading and chemical analysis of nanodrugs

Loading amount of drug conjugated on nanomaterials was quantified by an analytical technique which features an absorption spectrum. The differences in absorbance peak between the loaded drug (DOX) and base nanomaterial at a specific wavelength corresponded to the amount of loaded drug. To verify all analytical calculations obtained from analytical procedures, the weight differences between drug-loaded nanomaterials and unloaded base nanomaterials (i.e., carboxylated CNT and PEG coated CNT) were directly measured and the reciprocal coincidences of weight (UV-vis and balance) were compared (Fig. 1d and Figs S3 and 4). The weight ratio of loaded drug for the three types of nanodrugs was approximately 112%, 31.4% and 17.0% for covalent conjugation-DOX, PEG coat-DOX, and encapsulation-DOX, respectively (Figure S5). The chemical changes induced by the loaded drugs on the nanomaterials were identified by Fourier transform infrared spectroscopy (FTIR). Results revealed a coincidence of several IR peaks between the free drug and loaded nanodrugs (i.e., for covalent conjugation and PEG coat) (Figure S6a-b).

### Nanodrug release at extracellular and intracellular condition

Understanding the drug release profile in extracellular and intracellular physiological environments is a critical factor to understand the “switch on” mode of anti-cancer molecules to activate drug release from the nanomaterial^24^.

**Figure 2:**
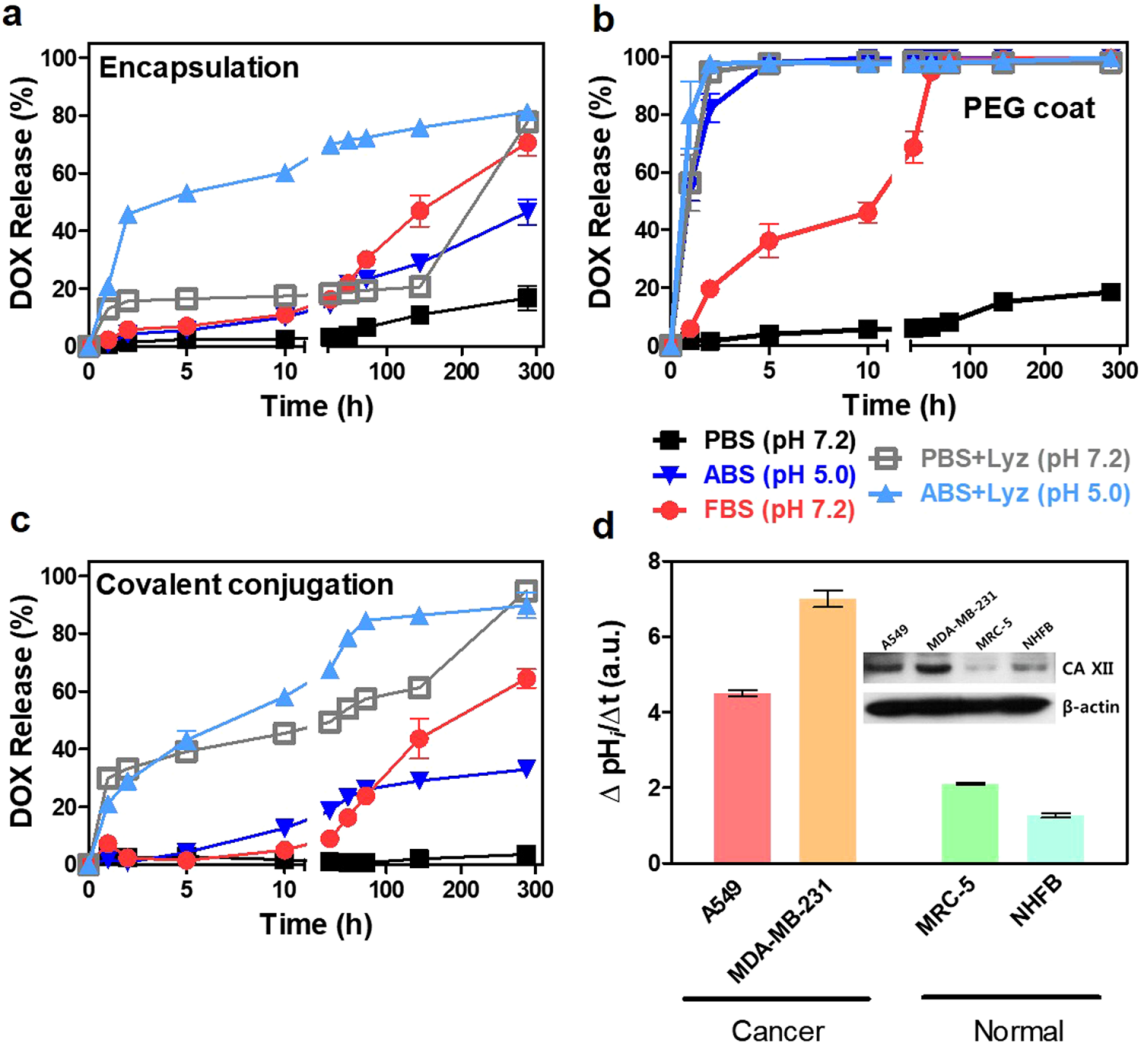
Extracellular and intracellular drug release analysis. (**a–c**) Released DOX by the different types of nanodrugs (i.e., covalent conjugation, liposomal encapsulation and PEG coat). **(a)** DOX release from encapsulation**, (b)** PEG coat and **(c)** covalent conjugation were analyzed in neutral (i.e., PBS), acidic (i.e., ABS), FBS (i.e., pH 7) and lysozyme-supplemented (i.e., both at pH 7 and pH 5) buffers up to 288 hrs. All data represent the mean ± SEM (n = 3). Most of the nanodrugs exhibited high drug stability in neutral PBS (pH 7.2) buffer. PEG coated drugs abruptly released (nearly 100% of the drugs), which detached from nanotubes in acidic buffer (pH 5.0 of ABS). In contrast, both covalent conjugation and liposomal encapsulation exhibited relatively stable drug release (i.e., less than 50% until 288 hrs) compared to PEG coat. PEG coated nanodrugs exhibited fast release (over 50% of the drugs were released within 10 hrs) in FBS buffer (10%, pH 7.2). **(d)** Western blot analysis for CA XII expression and measurements of acidification rate based on intracellular pH (pH_*i*_). Enhanced expression of CA XII proteins in A549 and MDA-MB-231 increased CO_2_/HCO_3_^−^ transporting machinery and, thus, facilitated intracellular acidification rate and provided more acidic intracellular conditions.

To examine the stability of drugs under extracellular conditions, drug release in neutral PBS (pH 7.2) buffer was analyzed and results showed all types of nanodrugs exhibiting high drug stability (e.g., liposomal encapsulation and PEG coat showed less than 20% drug release until 288 hrs) (Fig. 2a–c). In particular, covalent conjugation exhibited the most stable drug stability in PBS (Fig. 2c) (i.e., less than 5% release in PBS at 288 hrs). In contrast, DOX was abruptly released in acidic buffer (i.e., pH of 5.0 in acetate-buffered saline (ABS)) and almost 100% of the DOX detached from the PEG coated nanodrugs (Fig. 2b). Drug stability in a hemologic environment is important because the release of drugs from the nanocarrier prior to cell uptake will minimize the anti-cancer efficacy of nanocarriers in cancer cells. Buffer supplemented with blood enzymes (i.e., fetal bovine serum (FBS) 10% with pH = 7.2), was used to emulate the blood-like physiological conditions. In FBS-supplemented buffer (pH 7.2), PEG coat-DOX corresponded to a fast drug release, moreover 50% of conjugated drugs were released within 10 hrs (Fig. 2b). This suggested that the enzymes in FBS detached the DOX, even under neutral pH conditions. Both liposomal encapsulation and covalent conjugation showed identical release patterns until 288 hrs (Fig. 2a and c) and confirmed the increased stability of liposomal encapsulation and covalent conjugation compared to PEG coat.

While minimal drug release in the extracellular environment is generally favorable, drug transport to the specific intracellular organelles with intracellular environment is essential^30^. Intracellular-like conditions were generally represented by two major biochemical parameters: pH and the presence of active enzymes. Importantly, examining drug release in buffer containing the hydrolase enzyme and featuring a low pH can mimic acidic intracellular condition in cancer cells. To examine acidic condition in cancer cells, tumor-related carbonic anhydrase CA XII was investigated for tested cancer and normal cell lines. CA XII is generally expressed on the plasma membrane to regulate pH by mediating the reversible hydration of CO_2_ to HCO_3_^−^ to mediate acid-base balance^31^. Ectopically expressed CA XII is essential for the HCO_3_^−^ transport system with the recruitment of an anion exchanger as an acid loader^32^. In this study, enhanced expression of CA XII in A549 and MDA-MB-231 significantly increased CO_2_/HCO_3_^−^ transporting machinery and, thus, facilitated the intracellular acidification rate to, ultimately, render more acidic intracellular environments (Fig. 2d and Figure S7a–d). The CO_2_ was proposed as a major source of acidity in tumors^33^. In the presence of CO_2_, cancer cells may promote acidic intracellular environments by the regulation of CA XII and, in this manner, facilitate nanodrug release in acidic environments^33^.

The enzymatic cleaving process is generally initiated in the early/late endosomal stage and the density of the enzymatic proteins, lysozyme (Lyz), was greater during the lysosomal degradation stage. Most of PEG coat-based nanodrugs was released within 2 hrs by the emulated enzymatic environments (i.e., at both pH of 5.0 and 7.2) (Fig. 2b). The results confirmed that PEG coat nanodrugs evolve the LE stage from the EE stage were unstable, since enzymatic activity increases in LE. Liposomal encapsulation exhibited selective drug release in acidic enzyme environments (i.e., pH 5.0), but drug release relatively stable in neutral pH, enzymatic environments (i.e., pH 7.2) (Fig. 2a). Liposomal encapsulation exhibited more stable drug release than covalent conjugation in enzymatic environments with neutral pH, but exhibited identical drug release patterns to liposomal encapsulation in enzymatic environments with acidic pH (Fig. 2a and c). Thus, liposomal encapsulation might provide a pH-dependent switch mechanism for triggering drug release at the LE stage (or Lys stage) due to the dense collection of digestive acidic enzyme proteins in the vesicles. In conclusion, liposomal encapsulation and covalent conjugation were both stable, and sustain the conjugated drugs onto the nanomaterial until evolving Lyz degradation stage (i.e., pH under 5), whereas PEG coat was extremely vulnerable in both low acidic and enzymatic environments.

### Nanodrug uptake analysis

The intracellular pathway is highly associated with uptake types of cell penetration^34^ and, thus, the type of cellular uptake elicited by each type of nanodrugs was identified. First, it was determined whether the tested cancer and normal cells possessed the original ATP-assisted (i.e., dynamin-dependent) uptake markers. In this study, tested cancer cell lines exhibited positive clathrin and caveolin markers, but caveolin markers were negligible in normal cells (Figure S8). In contrast, macropinocytosis was positive for the tested normal cell lines examined in this study (Figure S8). Furthermore, the active drug uptake markers were evaluated for the various types of nanodrugs. Results indicated that free DOX showed no ATP-dependent uptake markers for all tested cell lines (i.e., both cancer and normal cells) (Figure S9a,b), as expected based on previous reports and supported by the evidence of diffusive intake of drug molecules (non-ATP assisted uptake)^35, 36^. Both liposomal encapsulation and PEG coat exhibited moderate clathrin-dependent uptake for both cancer and normal cell lines (Figure S9a,b). Notably, greater clathrin uptake was observed in covalently conjugated DOX (Figure S9a). Compared with liposomal encapsulation and PEG coat, covalently conjugated nanodrugs greatly activated clathrin uptake (Figure S9a). The overall uptake levels in normal cell lines were less than that of cancer cell lines (Figure S9a,b). It is worth noting that positive caveolin uptake markers in cancer cells were not observed with the tested nanodrug (Figure S9a) and this suggested that the size of the nanodrugs was not adequate (i.e., >50 nm) for triggering caveolin uptake^10^. The appropriate inhibitor concentration was determined by ensuring 80–90% of cell viability (i.e., uptake inhibitor toxicity) for the tested inhibitors (i.e., 5-(N-Ethyl-N-isopropyl) amiloride (EIPA), genistein (GEN) and chlorpromazine (CPZ)) to ensure that the inhibitors do not interfere with the uptake ability for the tested cells (see Figure S10).

### Intracellular pathways of cancer and normal cells

**Figure 3:**
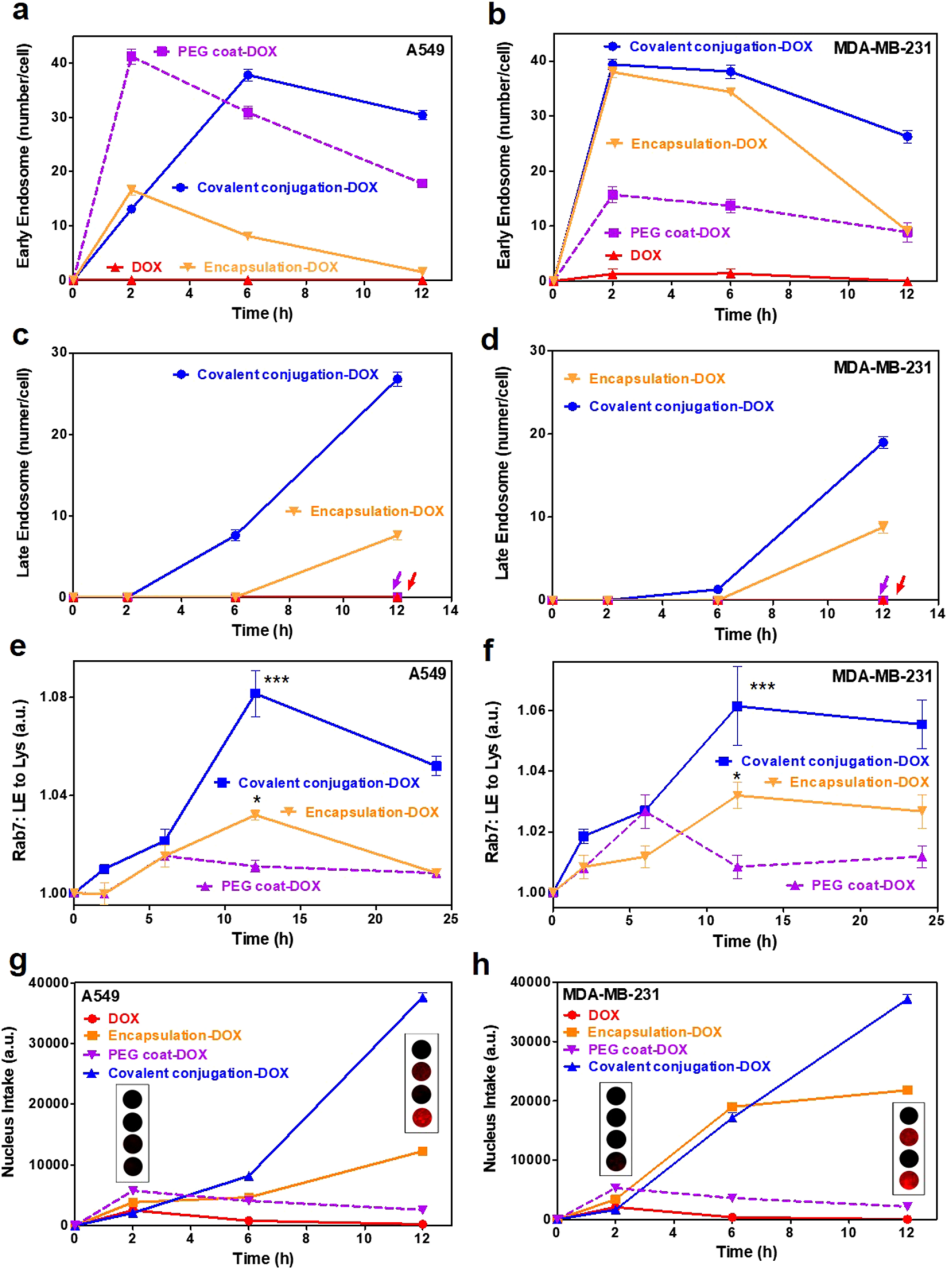
Intracellular trafficking (EE, LE, Lys and nucleus intake) of nanodrugs. (**a–d**) Quantification of time-dependent co-localization from confocal microscopy images. Time-dependent co-localization of each nanodrug and EE for (**a**) A549 and (**b**) MDA-MB-231 cells. Time-dependent co-localization of each nanodrug and LE in (**c**) A549 and (**d**) MDA-MB-231 cells. Only covalent conjugation (blue) and liposomal encapsulation (orange) developed from EE to LE. The formation rate and number of LE vesicles in covalently conjugated samples was higher and greater, respectively, than that of liposomal encapsulation. Rab7 expression profile (indicative of LE to Lys) in covalent conjugation (blue) and liposomal encapsulation (orange) samples showed greater Lys levels from EE, whereas results from other types of nanodrugs (i.e., purple: PEG coat) were insignificant for both **(e)** A549 and (**f**) MDA-MB-231 cells (until approximately 24 hrs). Time dependent fluorescence intensity corresponding to DOX intake within the nucleus of (**g**) A549 and (**h**) MDA-MB-231 cells for the various types of nano-conjugations. Both covalent conjugation and liposomal encapsulation induced significant difference of DOX fluorescence in the nuclei after 10 hrs. EE and LE data represents the mean ± SEM (n = 10). Rab7 and nucleus intake data represents the mean ± SEM (n = 5). *, ** and *** correspond to p < 0.05, p < 0.01 and p < 0.001, respectively, as compared with PEG coat-DOX (i.e., purple).

After clathrin (major) and/or macropinocytosis (minor) uptake by cancer cells, the drug delivery behavior differed amongst the various types of nanodrugs. Specifically, covalent conjugation, encapsulation, and PEG coat showed higher levels of EE (based on confocal image-based counts of EE vesicles per cell) on A549 and MDA-MB-231 compared to DOX (Fig. 3a,b and Figure S11a). The formation of EE vesicles reached a maximum within a few hrs (i.e., 2–6 hrs) (Fig. 3a,b). Similar trends were observed in MDA-MB-231 and the maximum EE counts were again observed at 2-6 hrs (Fig. 3b and Figure S11b). Both A549 and MDA-MB-231 showed the identical formation and disappearance patterns of EE after a few hours (Fig. 3a,b).

The LE stage generally exhibits lower intracellular pH (pH_*i*_) and a high concentration of acidic hydrolase enzymes. In this study, only covalent conjugation and liposomal encapsulation developed to the LE stage. The observed formation and number of LE vesicles were faster and greater in numbers by covalent conjugation than liposomal encapsulation (i.e., LE (Fig. 3c,d). However, fluorescence intensity from encapsulated DOX may be diminished by liposomal encapsulated lipid bilayer. The obtained LE results explicitly indicated that most of the PEG coat did not develop to the LE stage (Fig. 3c,d) but may have entered the RE stage and released into cytosol after the EE stage. A greater amount of low-pH vesicles (lysotracker analysis, Figure S12) and increased RE (for Rab11 analysis, see the extracellular pathways section) with PEG coated nanodrugs supported this evidence. In normal cells, majority of nanodrugs were developed from EE except DOX treated cells (Figure S13a–d). However, no significant transition from EE to LE was observed. Overall, the formation rate and number of EE vesicles was significantly lower than that of cancer cells (Fig. 3a–d and Figure S13a–d).

To examine the transition from the LE to the Lys stage, it is critical to evaluate the Rab7. As previously reported, Rab7 is a dominant protein during the mature stage of LE, which fuse together and become Lys^37^. In this study, maximum Rab7 levels were observed at 12 hrs with covalent conjugation and liposomal encapsulation, whereas PEG coat showed insignificant values during the inspection time (i.e., until 24 hrs) (Fig. 3e,f). The Rab7 trends of covalent conjugation and liposomal encapsulation were identical for the tested cancer cells (i.e., both A549 and MDA-MB-231) (Fig. 3e,f). At the Lys stage, the greater presence of enzymatic hydrolase was indicative of subsequent drug release from covalently conjugated and liposomal encapsulated nanodrugs, as previously identified by the analysis of released drug (Fig. 2a and c). In contrast, Rab7 expressions between tested nanodrugs were non-significant for both MRC-5 and NHFB normal cells (i.e., until 24 hrs) (Figure S13e,f).

Furthermore, no DOX transportation from LE to the trans-Golgi network (TGN) was observed with covalent conjugation and encapsulation, as determined by Rab9 analysis (Figures S14a,b and S15a,b). This suggested that covalent conjugation and liposomal encapsulation did not transport DOX to the TGN, but solely developed into Lys (low-density lipoproteins (LDL) was used as positive marker in the Rab9 analysis) (Figures S14a,b and S15a,b).

The Rab2 proteins are activated during trafficking from the Golgi to the endoplasmic reticulum (ER)^37^. Rab2 protein analysis in this study supported the evidence that DOX molecules intrinsically do not transport from the Golgi to the ER and instead directly diffuse to nucleus (Figures S14c,d and S15c,d). DOX fluorescence intensity in the nucleus increased over the observed time points and significant expression was observed after 10 hrs on covalent conjugated and encapsulated nanodrugs (Fig. 3g,h). Thus, it was concluded that the majority of DOX was released from the LE and Lys and directly diffused into the nucleus of cancer cells^38^. In normal cells, covalent conjugation increased DOX fluorescence in the nucleus of MRC-5 (Figure S15e) whereas both covalent conjugation and liposomal encapsulation increased DOX intake in the nucleus of NHFB after 10 hrs (Figure S15f).

Altogether, the Rab7, Rab9, and Rab2 results indicated that DOX diffused to the cancer nuclei through the LE to Lys stages, not through the TGN to ER pathway (based on Rab9 and Rab2 analysis). In this regard, covalent conjugation and liposomal encapsulation are more advantageous for transporting drugs to the nucleus, whereas PEG coat was advantageous for transport drugs (DOX) to the cytosol.

### Mitochondrial accumulation of nanodrugs

**Figure 4:**
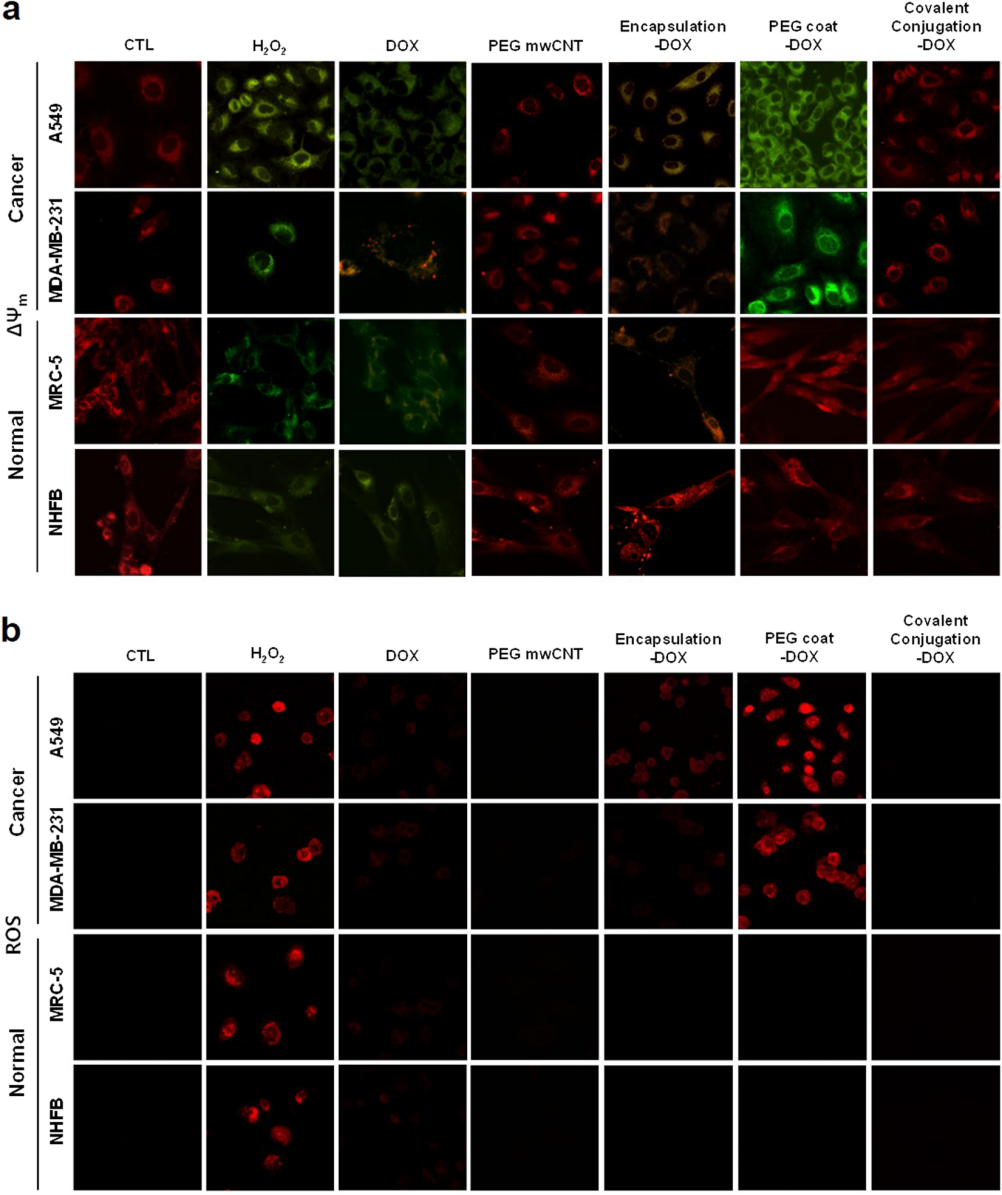
Changes in mitochondrial membrane potential (ΔΨ_m_) and Fluorescent ROS intensity (DHE) due to nanodrugs. (**a**) JC-1 staining showed depolarized mitochondria (green, J-monomer) and polarized mitochondria (red, J-aggregate) membrane potentials after 24 hrs. Hydrogen peroxide (H_2_O_2_) was used as a positive control. PEG coated conjugations of DOX significantly influenced mitochondrial membrane potential in cancer cells (i.e., both A549 and MDA-MB-231), whereas normal cells (i.e., MRC-5 and NHFB) did not show any notable changes (e.g., NHFB) in membrane potential. (**b**) Confocal microscopy images indicated positive ROS after 24 hrs. ROS induced by the various types of nanodrugs showed identical trends with respect to ΔΨm. PEG coated nanodrugs (i.e., DOX) induced elevated levels of ROS for all cancer cells. Interestingly, covalent nano-conjugation did not invoke any ROS.

Drug targeting to the mitochondria can destroy the energy generation units in cancer cell and, thus, induce apoptosis^39, 40^. In this study, the authors identified that choosing the appropriate type of nanodrug can enhance the probability of drug accumulation in the mitochondria. To analyze selective mitochondria targeting, changes to the mitochondria membrane potential (JC-1) due to drug interaction (i.e., with free DOX) were examined (Fig. 4a and Figure S16a). PEG coated conjugation with DOX significantly increased changes to the mitochondria membrane potential of cancer cells (A549 and MDA-MB-231) (Fig. 4a and Figure S16a). This was interpreted that the drugs released from the EE into the cytosol resulted in accumulation in the mitochondria. For normal cell lines (both MRC-5 and NHFB), less uptake and a lower amount of EE vesicles resulted in negligible mitochondria accumulation, whereas the release of free DOX reached the mitochondria via the cytosol (Fig. 4a and Figure S16a).

The induced ROS intensity showed identical trends with △Ψ_m_ for the various nanodrugs (Fig. 4b and Figure S16b). The most interesting finding was the non-interference of Ψ_m,_ as well as the ROS intensity with covalent conjugation (Fig. 4b and Figure S16b). In contrast with free drug, PEG coat and liposomal encapsulation, covalent conjugation did not influence mitochondrial function (Fig. 4b and Figure S16b). This confirms that most nanodrugs with covalent conjugation completely entered the EE, LE and Lys, and, thus, did not have the chance for drug release in the cytosol.

In conclusion, PEG coat is advantageous for drug delivery to the mitochondria, whereas covalent conjugation is highly effective for drug delivery to the nucleus (see Fig. 6). Previous mitochondrial targeting was achieved by highly positive charged (i.e., above 18 mV) nanodrugs^41^. However, the observed electrical potential (1.19 mV) of PEG coat-DOX nanodrug in this study (Fig. 1b) was not on a comparable order of positive charges to attract mitochondria. Liposomal encapsulation was effective for drug delivery to both the nucleus and mitochondria (Figs 3g,h and 4), but not advantageous to drug selective for the nucleus or the mitochondria (see Figs 3–4 and 6). The obtained results stressed the importance of choosing the appropriate type of nanodrug (i.e., liposomal encapsulation, PEG coat or covalent conjugation) for delivering organelle-specific active chemotherapeutic agents into the desired intracellular organelles.

### Extracellular pathways (nanodrugs efflux, clearance, recycling endosomal exocytosis and exosome analysis)

**Figure 5:**
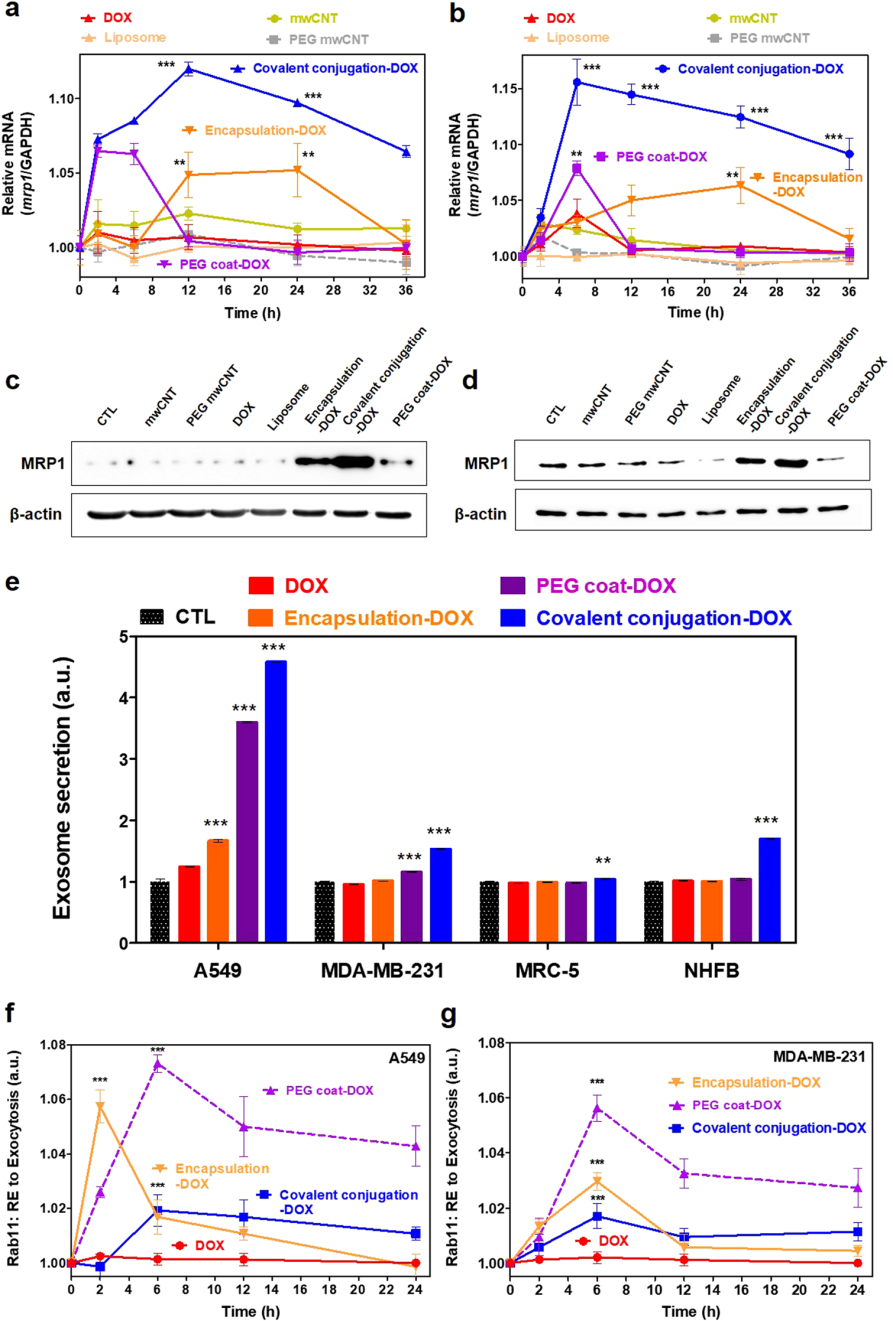
Extracellular pathway (efflux, exosomes and RE exocytosis) of nanodrugs. Time-dependent cellular removal of nanodrugs by efflux pumps (*mrp-1*) in (**a**) A549 and (**b**) MDA-MB-231 cells. Although PEG coat, liposomal encapsulation and free drugs exhibited relatively short time periods of efflux upregulation, covalent conjugation (blue line) exhibited prolonged upregulation (i.e., approximately 36 hrs) of the cancer efflux system (*mrp-1* gene expression). Western blot analysis of MRP-1 expression after treatment (24 hrs) of nanodrugs in (**c**) A549 and (**d**) MDA-MB-231 cancer cells. (**e**) Secreted exosomes from cancer cells (e.g., A549 and MDA-MB-231) and normal cells (e.g., MRC-5 and NHFB) showed significant in nanodrug samples with covalent conjugation (blue) compared to liposomal encapsulation (orange) and free DOX (red) after 24 hrs. Time dependent analysis of Rab11 expression (indicative of RE to exocytosis). PEG coated conjugation (i.e., DOX: purple marker) exhibited increased Rab11 levels compared to covalent conjugation (blue) and liposomal encapsulation (orange) in (**f**) A549 and (**g**) MDA-MB-231 cell lines. Efflux and exosome data represents the mean ± SEM (n = 3). RE exocytosis data represents the mean ± SEM (n = 5). *, ** and *** correspond to p < 0.05, p < 0.01 and p < 0.001, respectively, as compared with free DOX (i.e., red).

Uptaken drugs in cancer cells first encounter efflux pumps. Activation of the drug efflux system is one of the major characteristics of cancer resistivity^42^ and, thus, examining drug efflux-associated genes and proteins expression can provide an understanding how cancer cells response with the uptaken nanodrugs. From *p-gp* and *mrp-1*, only mrp-1 mRNA expression was detectable in A549 and MDA-MB-231 cells (Figure S17). As was the case with intracellular pathways, different types of nanodrugs demonstrated different reactions to the cancer efflux and exocytosis. It is worth noting that covalently conjugated and some portion of liposomal encapsulated drugs exhibited prolonged upregulation of cancer efflux (i.e., *mrp-1*) within the first 36 hrs via prolonged intracellular trafficking, such as with LE and Lys, whereas cytosol-released drugs (i.e., PEG coat, liposomal encapsulation and free drugs) exhibited relatively short time activation of efflux upregulation (Fig. 5a–d). Liposomal encapsulation also exhibited slower and slight upregulation compared with covalent conjugation, but recorded notable *mrp-1* gene expression (i.e., during 12–36 hrs) (Fig. 5a–d and Figure S18a,b) and, thus, indicated that the efflux of cancer cells continue to eject for prolonged intracellular trafficking, such as with LE and Lys. It is also worth noting that the efflux of liposomal encapsulation (as determined by *mrp-1* analysis) was activated relatively late compared to covalent conjugation (Fig. 5a,b). This late *mrp-1* appearance might be related to the delayed appearance of LE with liposomal encapsulation (based on previous LE analysis, see Fig. 3c,d).

Exosomes were recently considered an important marker for cellular elimination of uptaken nanodrugs (or nanoparticles) and, hence, a major marker of a non-classical exocytic secretary pathway^43^, usually followed by the MVB/LE formation after the EE stage^44^. Thus, lysosomal degradation and exosomal release are continual markers during the elimination of uptaken nanodrugs. Although efflux pumps and exosomal release are both active markers of elimination, the main difference between efflux and exocytosis is the secreted object. Most exosomes contain cell-specific proteins and CDs that serve as biomarkers of the cell’s status^45^, whereas efflux pumps mainly eject uptaken drugs from cytosol^46^. Based on the exosomal secretion in this study, the amount of exosomes significantly increased with covalent conjugation compared to other types of nanodrugs (both cancer and normal cells) (Fig. 5e). The amount of exosomal secretion increased in the following order: free drug < liposomal encapsulation < PEG coat < covalent conjugation for both A549 and MDA-MB-231 cancer cell lines (Fig. 5e). The increased exosome count with covalent conjugation in NHFB (i.e., normal cells) was considered a consequence of the increased amount of LE (but negligible transition to the Lys stage, see Figure S15f), which may result in more exosome secretion. Overall, the exosomal secretion analysis should simultaneously consider the results of both the LE and low-pH-sensitive markers testing (i.e., from lysotracker, Figure S12). Among the various types of nanodrugs, covalent conjugation showed only significant exosomal secretion in both cancer and normal cells (Fig. 5e).

The extracellular elimination of uptaken nanodrugs through RE exocytosis was also investigated by examining Rab11 expression. Rab11 is a well-known recycling endosomal compartment protein, and a key regulator of extracellular cargo transport to the cell surface and strongly associated with the efflux of uptaken drugs^47^. As expected, PEG coated nanodrugs transferred from the EE to the RE compartments without reaching to the LE or Lys stage, as identified by Rab11 analysis (Fig. 5f,g). The majority of PEG coated nanodrugs steadily released to the extracellular membranes by RE exocytosis in cancer cells (Fig. 5f,g). In contrast, Rab11 analysis showed a lower amount with covalent conjugation (Fig. 5f,g). From this, it was interpreted that most of the nanodrugs with covalent conjugation transitioned to the LE and Lys from the EE and, thus, no significant Rab11 expression was observed in cancer cells, as compared with PEG coat (Fig. 5f,g). The Rab11 intensity of encapsulated nanodrug samples also significant differed from that of covalent conjugation (A549), where the level for encapsulated nanodrugs lied between that of PEG coat and covalent conjugation (MDA-MB-231) (Fig. 5f,g).

In conclusion, a substantial amount of PEG coated drug (i.e., DOX) underwent exocytosis through the RE pathway without entering acidic lysosomal degradation. DOX clearance test of covalent conjugation showed the least amount of effluxed DOX from cancer cells (e.g., both A549 and MDA-MB-231). Specifically, 25~40% of the total amount of DOX still accumulated in the intracellular region over 36 hrs (Figure S19a,b). In contrast, free DOX and encapsulation-DOX were more quickly eliminated (100%) from the cells, compared with PEG coat (10% remaining) and covalent conjugation-DOX (40% remaining) until 36 hrs (Figure S19a,b). A higher retention of DOX in cancer cells (i.e., low clearance rate) may invoke continued upregulation of the *mrp-1* efflux system to remove over the remaining drugs (Fig. 5a,b).

Although cancer cells clearly demonstrated different patterns of efflux for the tested nanodrugs, normal cells (i.e., both MRC-5 and NHFB) exhibited no significant difference amongst the various types of nanodrugs groups after 10 hrs (Figure S19c,d). This indicated that the tested normal cell lines do not possesses enough LE and Lys in the intracellular environment.

**Figure 6:**
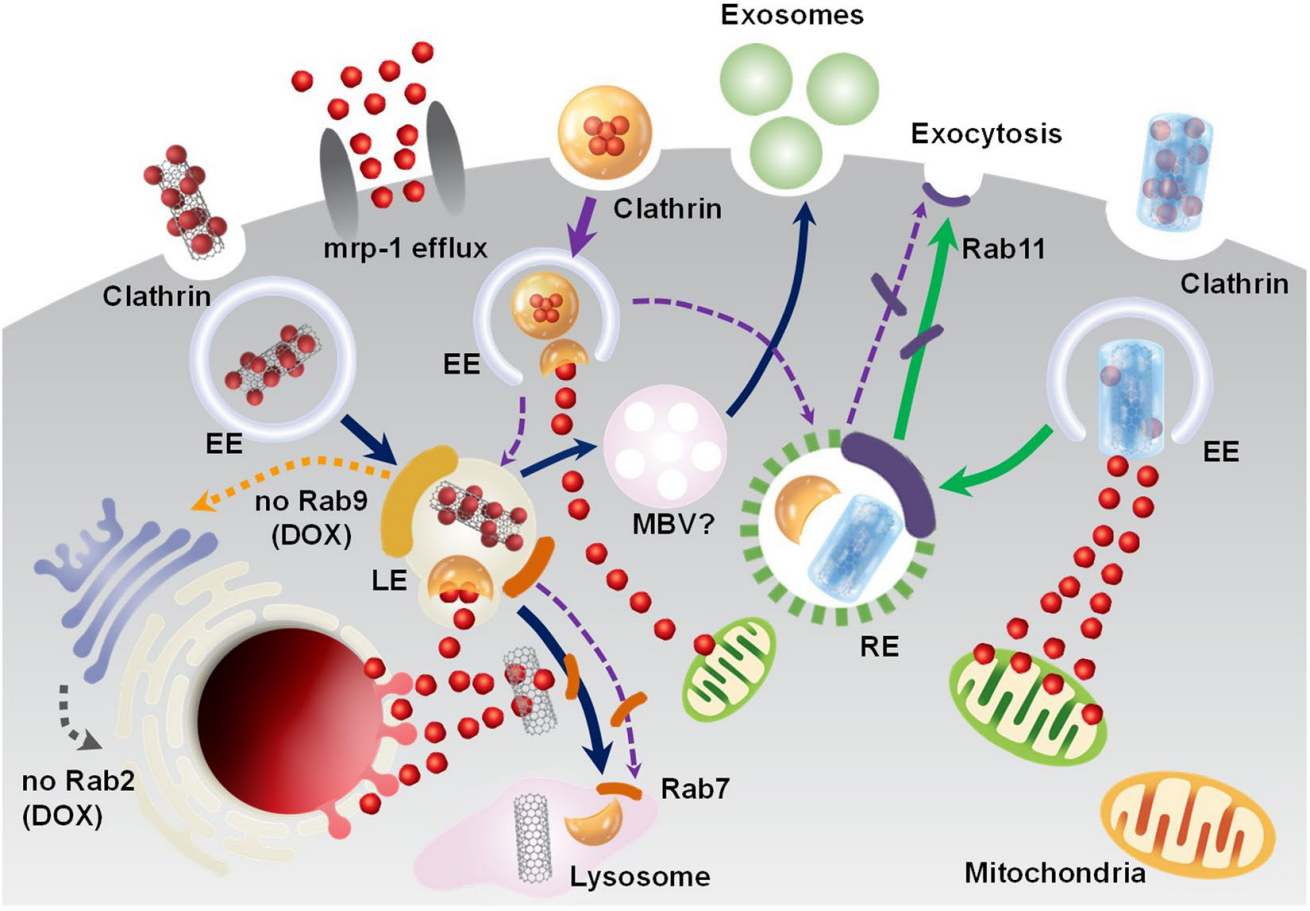
Schematic illustration of intra- and extracellular trafficking dynamics of covalent conjugation, PEG coat and encapsulated nanodrugs after cellular uptake (cancer).

In conclusion, efflux activation, exosome exocytosis, RE-assisted exocytosis, drug clearance (or efflux amount) and the associated intracellular trafficking (EE, LE and Lys) are closely related and influenced multiple types of extracellular pathways, directly or indirectly resulted in various aspects of extracellular pathways (Fig. 6). Importantly, the trends of extracellular pathways were clearly different depending on the types of nanodrugs.

### Differential apoptosis, cytotoxicity and the selective influence of covalent anti-EGFR conjugation on cancer and normal cells

**Figure 7:**
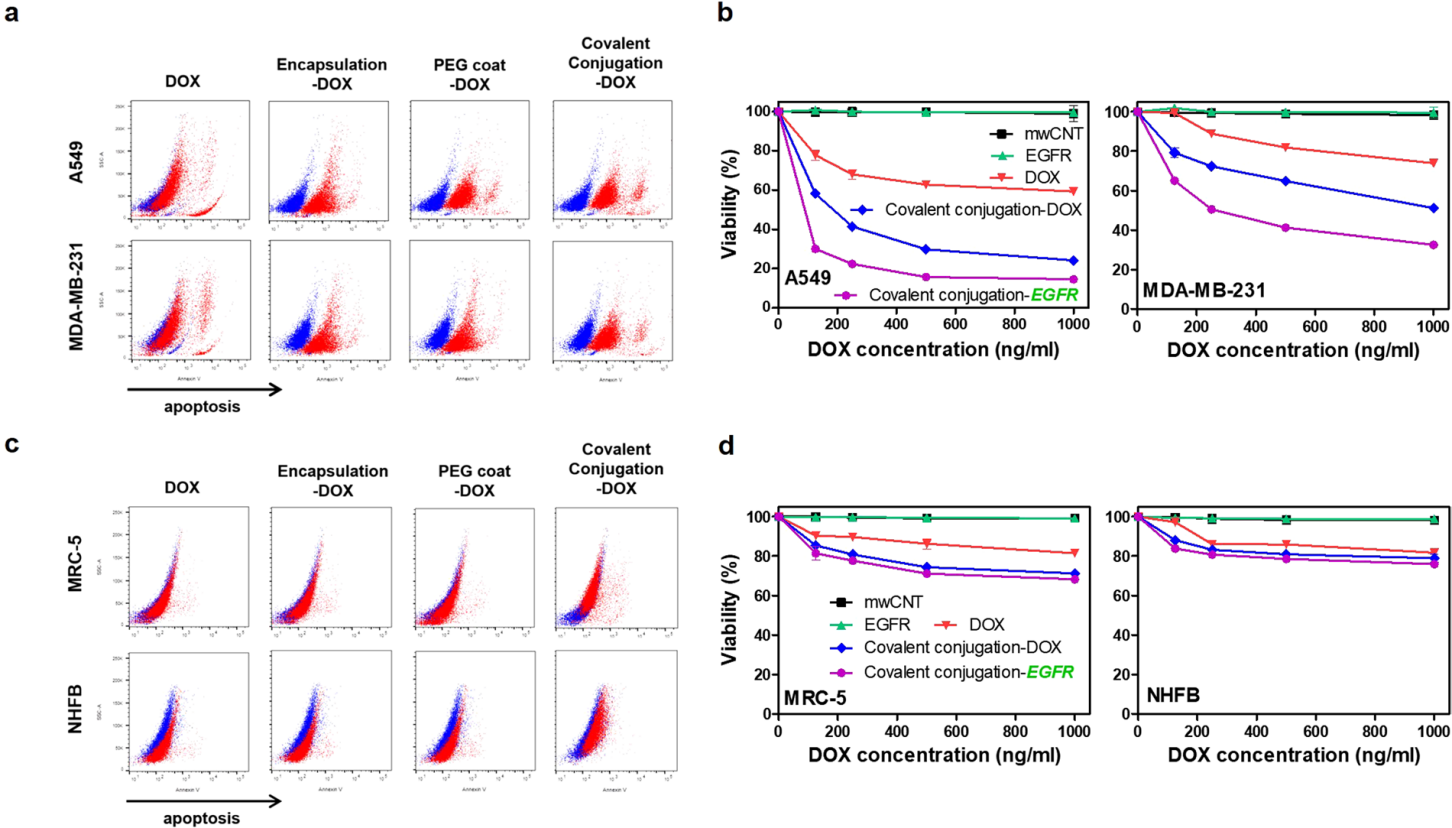
Differential cell apoptosis and cytotoxicity on cancer and normal cells by various types of nanodrugs. (**a**) Blue and red markers correspond to control and drug-treated cells, respectively. A549 and MDA-MB-231 cells were stained with annexin V-Pacific blue. After 24 hrs of treatment with various types of nanodrugs, samples were analyzed by FACS. Encapsulation-DOX, PEG coat-DOX and covalent conjugation-DOX (red) showed significant apoptosis compared to controls (blue) (i.e., untreated cells). (**b**) MTT results of cancer cells (A549 and MDA-MB-231) by treatment of EGFR conjugated nanodrug. The addition of anti-EGFR conjugation (purple, covalent conjugation-*EGFR*) maximized the cytotoxicity compared with non-anti-EGFR nanodrugs (blue, covalent conjugation-DOX). (**c**) MRC-5 cells and NHFB showed statistically negligible differences in apoptosis for the various types of nanodrugs compared to controls (blue) (i.e., untreated cells). (**d**) MTT results of normal cells (MRC-5 and NHFB) by treatment of EGFR conjugated nanodrug. The addition of anti-EGFR conjugation (purple, covalent conjugation-*EGFR*) maximized the cytotoxicity compared with non-anti-EGFR nanodrugs (blue, covalent conjugation-DOX). All MTT data represent the mean ± SEM (n = 5).

Apoptosis is a biochemical process of programmed cell death^48^. In this study, various types of nanodrugs (i.e., covalent conjugation, PEG coat and liposomal encapsulation) yielded different apoptotic patterns for cancer cells (Fig. 7a and Figure S20). In short, most of the investigated nanodrugs elicited greater apoptosis and cytotoxicity for cancer cells than normal cells (Fig. 7a and c and Figures S20 and 21). The obtained results may be attributed to the different (innated) uptake activity and intracellular activity observed in cancer cells, such as the increased amount of endosomal and lysosomal vesicles that can transport the uptaken nanodrug to the cancer nucleus (i.e., covalent conjugation and encapsulation) or mitochondria (i.e., PEG coat) (Fig. 6). Specifically, covalent conjugation corresponded to the highest apoptosis, as well as greater cytotoxicity in cancer cells (Fig. 7a, Figures S20 and S21a). This was expected since covalent conjugation-DOX was highly effective for DOX delivery to the nucleus (Fig. 3g,h). In contrast, the efficacy of PEG coat (DOX) in cancer cells stemmed from increased mitochondrial damage by invoking ROS (Fig. 4a,b and Figure S16a,b). In contrast, normal cells showed lower cytotoxicity for the various nanodrugs investigated in this study (Figure S21b). This agreed well with the data presented earlier, which showed lower uptake activity, less endosomes (i.e., both EE and LE) and Lys for normal cells, as compared with cancer cells (see Fig. 3a–f, Figures S9a,b and S13a–f). The relatively small amount of endosomal and lysosomal vesicles did not appreciably elicit apoptosis and cytotoxicity in normal cells (Fig. 7c, Figures S20 and S21b). The different impact that the nanodrugs had on cancer versus normal cells highlights the importance of nanodrug design, on not only cancer but also normal cells.

Furthermore, the most efficient nanodrug for drug delivery to nucleus, covalent conjugation, was further investigated with the addition of anti-epidermal growth factor receptor (EGFR) antibodies (Fig. 7b,d). Docking anti-EGFR on the covalent conjugation nanodrugs not only increased the targeting probability to the outer cancer membrane, but also inhibited the activation of cell growth and proliferation in cancer cells by blocking EGFR^49^. The difference of cancer and normal cell viability was significant, such as, 28%, 62% (A549 and MDA-MB-231), 82% and 85% (MRC-5 and NHFB) at 100 ng/ml (Fig. 7b,d). It is demonstrated that anti-EGFR conjugated nanodrugs only promoted selective cytotoxicity in cancerous, but not normal cells (Fig. 7b,d and Figure S21c). In conclusion, adding covalently conjugated anti-EGFR maximized the cytotoxicity of cancer cells without eliciting toxicity in normal cells (as compared with non-anti-EGFR nanodrugs). As such, covalent anti-EGFR conjugation (biochemical) and the appropriate choice of nanodrug conjugation type (biosynthetic) are both influential factors for selectively controlling cancer cell cytotoxicity without elevating toxicity to normal cells.

## Conclusion

Clinical approval of anticancer nanodrug has been limited due to safety issues, such as renal clearance (reticuloendothelial system (RES)) and associated immune-response. In organic materials, such as gold, silica and carbon-based nanomaterials raised concerns in this regard and, thus, only few biodegradable materials including liposome and PEG polymers were authorized by FDA. However, if there is no substitute for inorganic nanomaterials in terms of anticancer drug efficacy, clinical use of inorganic nanomaterials need to be reconsidered under use of ultra-low concentration to avoid side effects by immunotoxicity.

In this regard, to increase the anticancer efficacy, this study comprehensively examined the role of nanodrug design (i.e., covalent conjugation, PEG coat and liposomal encapsulation) on the cellular uptake pathway, intracellular and extracellular dynamics, subcellular localization, and cytotoxicity for cancer and normal cells. The choice of nano-conjugation types (i.e., amide covalent bond, PEG coat, and encapsulation) corresponded to unique intracellular and extracellular dynamics, enabling selective subcellular targeting, and, ultimately, selective cytotoxicity in cancer, but not normal cells. A thorough understanding of the intracellular dynamics of various types of nanodrugs can maximize intracellular targeting efficacy and can minimize unwanted cytotoxicity in normal cells.

## Materials and Methods

### Materials preparation

Purified multi-walled carbon nanotubes (mwCNTs) (900–1260, SES, USA) and PEG-amine (PEG, MW = 5 kDa, NOF, Japan) were used to generate carboxylated and PEG coated mwCNTs (PEG coat). Carboxylated mwCNT (mwCNT) and PEG were synthesized according to previously described methods^50^. DOX (44583, Sigma), a commonly used chemotherapy agent to treat a wide range of cancers and performs by blocking DNA replication^51^. To conjugate on carboxylated and coat PEG on mwCNTs, DOX (covalent conjugation-DOX and PEG coat-DOX) was used as base anticancer drug. Liposome and DOXOVES^®^ (liposomal encapsulation-DOX) were purchased from FormuMax Scientific, Inc. (Palo Alto, CA, USA).

### Conjugation methods and physical characterization of nanodrugs

The carboxylate mwCNTs were dispersed in 2-morpholinoethanesulfonic acid (MES) buffer (50 mM, pH 6.0, M3671, Sigma) by sonication (JEIOTECH. CO., Daejeon, Korea) for 5 min. Next, N-hydroxysuccinimide (NHS, 130672, Sigma) diluted in MES buffer (50 mM at pH 6.0) to a concentration of 400 mM was added to the carboxylated mwCNT solution and stirred with a vortex mixer for 5 min. The pH of the MES buffer was maintained at 6.1. N-(3-Dimethylaminopropyl)-N′-ethylcarbodiimide hydrochloride (EDC) (300 mM in MES buffer solution, E6383-5G, Sigma), then added to the solution and stirred for 30 min on a platform rocker. The mixture was then dispensed into filter tubes (UFC910024, Ultracell^®^-100K, 100 kDa, Millipore, Billerica, MA, USA), centrifuged at 4,000 rpm for 15 min, and rinsed with 50 mM MES buffer at least five times. The EDC-linked mwCNT-COOH solution was then mixed with DOX (1225703, Sigma) at a weight ratio of 1:1 in MES buffer (with the pH maintained at 6.0). The mixture was agitated at 4 °C overnight. The mwCNT-DOX conjugated suspension (covalent conjugation-DOX) in MES was centrifuged and filtered (Amicon YM-100K with 4000 rpm) more than 3 times to remove unconjugated drugs. Last, covalent conjugation-DOX were dispersed and stored in PBS (10010-023, Gibco, Waltham, MA, USA). To measure the concentration of DOX and mwCNTs, UV-vis (Libra S50, Biochrom, Cambridge, UK) was performed and the adsorption analysis was performed at 490-nm-wavelength. The weight percentage of covalently-linked DOX on mwCNT was determined by measuring the difference in absorbance signal intensities at 490 nm between covalent conjugation-DOX and unconjugated DOX. Weight density of the solutions was extrapolated from linear standard curves of DOX and oxidized mwCNTs, respectively.

To coat the mwCNTs on PEG by non-covalent conjugation, carboxylated mwCNTs (oxidized by acidic treatment) were mixed with PEG in de-ionized water, sonicated for 30 min and dispensed into filter tubes (Amicon YM-50, 100 kDa, Millipore) for centrifugation at 4,000 rpm for 10 min. The PEG coated mwCNT solutions were then washed with MES buffer at least three times prior to mixing with DOX in MES buffer. The mixtures were then kept at room temperature (RT) overnight with shaking. The PEG coated mwCNT-DOX (PEG coat-DOX) in MES buffer was filtered (Amicon YM-50, 100 kDa, Millipore) by centrifugation at 4,000 rpm at least 3 times to remove unconjugated DOX. Last, PEG coat-DOX was mixed with PBS (pH 7.2). The percentage of non-covalently attached DOX on PEG coated mwCNTs was determined by measuring the weight difference between PEG coat-DOX after at least two days in a vacuum oven at 60 °C. The particle size and electric potential were analyzed (Zetasizer Nano, Malvern, UK) to determine the size distribution profile and electric potential of PEG coat-DOX in PBS. Samples (1 mg/ml) completely dried by freeze drying at a temperature below −80 °C (Alpha 1–2 LD plus, Martin Christ, Germany) for 24 hrs and analyzed by FTIR (Fourier transform infrared spectroscopy). The attenuated total reflectance (ATF) mode of FTIR (Nicolet iS5, Thermo, Waltham, MA, USA) was used to analyze the drugs attached to mwCNTs and PEG coated mwCNTs. Each spectrum was obtained after 16 scans with a resolution of 4 cm^−1^. Cryogenic Transmission Electron Microscopy (Cryo-TEM, F20, Tecnai) was used to visualize covalently conjugated and PEG coated drugs on mwCNTs or liposomal encapsulated drugs on a copper grid (plasma etched before dipping into each nanoconjugated drug solution). Next, the prepared samples were instantly frozen in ethane and preserved in liquid nitrogen, using a plunge freezing technique in Vitrobot (FEI).

### Drug release analysis

To evaluate drug release, encapsulation-DOX, covalent conjugation-DOX and PEG coat-DOX were prepared in PBS (pH 7.2), acetate-buffered saline (ABS, pH 5.0) and PBS supplemented with 10% FBS (pH 7.2) with an initial concentration of 1 μg/ml. Drugs in PBS, ABS, and ABS with lysozyme were each subjected to gentle shaking and incubation at 37 °C. At each time point, the samples were centrifuged with a 50-kDa ultra-filter (Millipore) at 13,000 rpm for 5 min and 500 μl of supernatant was collected at 1, 2, 5, 10, 24, 48, 72, 144 and 288 hrs. The amount of DOX in the supernatant was analyzed by Victor X3 multi-label plate reader (Perkin Elmer, USA).

### Cell lines and cell culture

A549 (lung cancer cells, CCL-185™), MDA-MB-231 (breast cancer cells, HTB-26™) and MRC-5 (normal lung fibroblast cells, CCL-171™) were obtained from the American Type Culture Collection (Rockville, MD, USA). NHFB (normal dermal fibroblast cells, CC-2511) was obtained from the Clonetics (San Diego, CA, USA). Cancer cells and NHFB were maintained in Dulbecco’s modified Eagle’s medium (DMEM, 11995–065, Gibco, Waltham, MA, USA) supplemented with 10% fetal bovine serum (FBS, 16000–044, Gibco, Waltham, MA, USA) and 1% antibiotics (Invitrogen, Waltham, MA, USA). MRC-5 cells were maintained in Eagle’s Minimum Essential Medium (EMEM, 30–2003, ATCC) supplemented with 10% FBS and 1% antibiotics. All cells were cultured in a humidified incubator containing 5% CO_2_ at 37 °C.

### Endocytosis pathways identification (FACS)

All cells were seeded in a six-well plate at a density of 4 × 10^5^ cells/well and incubated overnight. The cells were pre-incubated with inhibitors (i.e., 5-(N-Ethyl-N-isopropyl) amiloride (EIPA), chlorpromazine (CPZ) and genistein (GEN), Sigma) for 30 min at 37 °C to examine macropinocytosis and the uptake of caveolin and clathrin. Before treatment with each endocytosis inhibitor, cell viability was checked using MTT assay to ensure that any cytotoxic effect was due to treatment with endocytosis inhibitors. The nanodrugs investigated (i.e., DOX, encapsulation-DOX, covalent conjugation-DOX and PEG coat-DOX) were then incubated with cells for another 2 hrs. The DOX concentration of all nanodrugs was 200 ng/ml. The concentrations of EIPA, chlorpromazine and genistein were 25 μM, 20 μM and 200 μM, respectively. Cells were then washed twice with PBS and suspended in 500 ml of PBS supplemented with 1% FBS. The DOX fluorescence obtained from single cell suspensions were evaluated using a BD LSR II flow cytometer (Becton Dickinson Immunocytometry Systems) and analyzed using FlowJo software (Ver. 10.1, FlowJo, LLC). Single cell suspensions treated with nanodrugs without inhibitors served as controls.

### Immunofluorescence analysis for intracellular trafficking (EE, LE)

Cells were cultured overnight on poly-D-lysine-coated coverslips in 24 well plates and treated with 200 ng/ml of DOX, encapsulation-DOX, covalent conjugation-DOX, PEG coat-DOX for the indicated time. Cells were fixed with 4% paraformaldehyde in medium overnight at 4 °C. Permeabilization was performed in PBS with 0.1% Triton X-100 for 15 min at RT. After a blocking period of 2 hrs with 1% bovine serum albumin (BSA, Generay Biotech Co., Shanghai, China) in PBS, cells were incubated with EEA-1 (polyclonal, ab2900, Abcam) and mannose 6-phosphate receptor (M6PR, a late endosome marker, 1:1000, ab2733, Abcam) antibodies overnight at 4 °C in the absence of light. After washing twice times with PBS, cells were incubated with Alexa Fluor 405 Goat Anti-Rabbit IgG (H + L) and Alexa Fluor 405 Goat Anti-Mouse IgG (H + L) (1:200 dilution factor, A11008, A21057, Molecular Probes) for 2 hrs at RT. Lastly, cells were mounted, visualized using confocal microscopy LSM700 (Carl Zeiss, Germany) and analyzed using the ZEN software. The number of co-localized (yellow color: co-localized section of DOX and intracellular vesicles) regions was counted in each cells (where a total 10 cells were analyzed for determining the number of EE or LE at a given time).

### Rab protein expression

All cells (5 × 10^3^ cells/well) were seeded in 96-well plates and cultured overnight. The media was replaced with drug-supplemented (e.g., encapsulation-DOX, covalent conjugation-DOX and PEG coat-DOX) media at 100 μl/well and incubated for the indicated time. Cells were fixed with 4% paraformaldehyde in medium overnight at 4 °C. Permeabilization was performed in PBS with 0.1% Triton X-100 for 15 min at RT. After a blocking period of 2 hrs with 1% BSA in PBS, cells were incubated with RAB2 (polyclonal, ab131568, Abcam), RAB7 (polyclonal, ab50533, Abcam), RAB9 (polyclonal, ab179815, Abcam) and RAB11 (polyclonal, ab3612, Abcam) antibodies overnight at 4 °C in the absence of light. After washing twice times in PBS, cells were incubated with Alexa Fluor 405 Goat Anti-Rabbit IgG (H + L) and Alexa Fluor 405 Goat Anti-Mouse IgG (H + L) (1:200, A11008, A21057, Molecular Probes) for 2 hrs at RT. Cells were washed twice times in PBS and fluorescence was measured with a microplate reader (VICTOR X3, PerkinElmer, Waltham, MA, USA) at 405 nm (excitation) and 460 nm (emission).

### Lysosomal tracking analysis

Cancer and normal cells (5 × 10^3^ cells/well in 96-well plates) were treated with 0.2 μg/ml of either DOX, encapsulation-DOX, mwCNT, PEG mwCNT, liposomes, covalent conjugation-DOX or PEG coat-DOX for 24 hrs. Cells were fixed with 4% paraformaldehyde in medium overnight at 4 °C. Permeabilization was performed in PBS with 0.1% Triton X-100 for 15 min at RT. After blocking with 1% BSA in PBS for 2 hrs, cells were incubated with Lysotracker (DND-22 Blue, Molecular Probes) to stain the acidic organelles for 2 hrs at RT in the absence of light. After washing three times with PBS, lysosome intensity in the cells was measured by a fluorescence micro-plate reader (VICTOR X3, PerkinElmer, Waltham, MA, USA) with an excitation at 405 nm and an emission of 460 nm.

### MRP-1 efflux analysis

In order to quantify the cellular expression of *mrp-1* genes, all RNA was extracted using TRIZOL reagent (Invitrogen, USA) at indicated times (i.e., 0, 2, 6, 12, 24 and 36 hrs) according to the manufacturer’s instructions. The absorbance (i.e., 260, 280 nm) were measured for RNA quantification using a Nanodrop 2000 UV-Vis spectrophotometer (Thermo, Waltham, MA, USA). Purity of the RNA in each sample was adjusted to an OD_260_/OD_280_ ratio of 1.8–2.0 and 2 μg of the RNA was reversely transcribed for synthesis of the first cDNA strand with M-MuLV Reverse Transcriptase (Promega, Fitchburg, WI, USA). For qPCR analysis, a thermal cycler CFX384 Real-Time PCR (Bio-Rad, Hercules, CA, USA) and a Light-Cycler-FastStart DNA Master SYBR Green I kit (Roche, Basel, Switzerland) were used according to manufacturer’s instructions. Briefly, 20 μl of reacted solutions were mixed with 1 μg of cDNA, 20 pmol of specific primers for measuring MRP-1 (forward, 5′-AGGTGGACCTGTTT CGTGAC-3′; reverse, 5′-TCCACCAGAAGGTGATC CTC-3′) and GAPDH ((forward, 5′-AGCTGAACGGGAAGCTCACT-3′; reverse, 5′-TGCT GTAGCCAAATTCGTTG-3′), and SYBR master mix (Product #04913850001, Roche, Basel, Switzerland). The cycle threshold (Ct) values were calculated using a CFX96 Real-Time PCR Detection System (Bio-Rad) software and the comparative Ct method (2^−ΔCt^ model) was used to calculate relative fold-changes in gene expression, which were normalized to the averaged GAPDH expression.

### Western blot analysis

Cells were rinsed twice with ice-cold PBS, and total cell lysates were gathered in 200 μl of lysis buffer (i.e., 20 mM Tris-HCl, 120 mM NaCl, 50 mM HEPES, 1% Triton-X, 1 mM EDTA, 2 mM sodium orthovanadate, 1 mM DTT, 10% glycerol, 0.02 mM PMSF, 1 mg/ml leupeptin, and 1 mg/ml aprotinin). The lysates were centrifuged at 12,000 rpm for 20 min at 4 °C, and the supernatant was collected. Proteins were electrophoresed using 8% SDS-PAGE, and then transferred to a PVDF membrane (Immobilon P, 0.45 pm mean pore size, Millipore Corp., Bedford MA). MRP-1 was assayed using anti-MRP-1 antibody (sc-7774, Santa Cruz Biotech, Dallas, TX, USA) and CA XII was assayed using anti-CA XII antibody (sc-374314, Santa Cruz Biotech, Dallas, TX, USA). The β-Actin was determined using anti-β-actin antibodies (sc-8432, Abcam). Immunodetection was performed using an enhanced chemiluminescence detection kit (34080, Thermo Scientific) and photographs of the protein bands were taken a LAS4000 ChemiDoc imager (Fujifilm, Japan). In case additional samples are displayed on the initial blots that are not relevant to this study, the images were cropped without additional modifications before analysis. Full-length blots are included in Figure S22.

### Extracellular DOX clearance analysis

To evaluate the extracellular release of nanodrugs from the cancer nucleus to the growth medium, the release of free DOX, encapsulation-DOX, covalent conjugation-DOX and PEG coat-DOX by cancer (i.e., A549, MDA-MB-231) and normal (i.e., MRC-5, NHFB) cells were tested. The cells were seeded on 96-well plates (at a density of 5 × 10^3^ cells/well) and cultured overnight. The growth medium was then aspirated and incubated with 100 μl of fresh growth medium that contained the drugs under investigation at a concentration of 200 ng/ml for 2 hrs. Following the incubation, cells were washed twice with fresh, drug-free culture medium and the cell-cultured supernatants was collected at the indicated times (i.e., 0, 1, 2, 6, 12, 24, and 36 hrs). Time course analysis for extracellular DOX fluorescence was measured in a multilabel plate reader (VICTOR X3, PerkinElmer, Waltham, MA, USA) at 470 nm excitation and 595 nm emission.

### Exosome isolation and quantitation

Cancer and normal cells (seeded at a density of 5 × 10^5^ cells/well in 6-well plates) were treated with 200 ng/ml of DOX, encapsulation-DOX, covalent conjugation-DOX and PEG coat-DOX for 24 hrs. Cells were rinsed with PBS to remove excess nanodrugs, exchanged with serum-free growth medium, and incubated for 24 hrs to permit exosome secretion. Following incubation, 4 ml of culture media was collected and exosomes were isolated using Total Exosome Isolation Reagent (Invitrogen, USA) in accordance with the manufacturer’s protocol. Briefly, isolated culture medium was centrifuged to remove cells and cellular debris (i.e., at 3,000 rpm for 30 min). The supernatant was mixed with 2 ml of exosome isolation reagent, and allowed to incubate at 4 °C for 12 hrs. Next, the mixture was centrifuged to collect the exosome pellet (at 13,000 rpm for 1 hr). The exosome pellet was resuspended in 100 μl PBS. The amount of exosome secretion was estimated by measuring the activity of esterase enzymes within the exosomes by the EXOCET exosome quantification kit (System biosciences, USA). The EXOCET assay is an enzymatic, colorimetric assay measured at a wavelength of 405 nm. Briefly, exosome samples (20 μl) were lysed by adding 80 µl of exosome lysis buffer directly to the exosomes suspended in PBS and incubated at 37 °C for 5 min to lyse the exosome membranes and solubilize the proteins. An exosome standard (SBI) diluted in PBS-B buffer were prepared for exosome standards at 0, 1.28 × 10^10^, 6.4 × 10^9^, 3.2 × 10^9^, 1.6 × 10^9^, 8 × 10^8^, 4 × 10^8^ counts. Standards and samples (in 50 µl volumes) were transferred to 96-well plates, mixed with EXOCET reaction buffer to a ratio of 1:1 v/v and incubated for 20 min at RT. The absorbance was read at 405 nm with a Biochrom Asys UVM 340 microplate reader. Quantitative results (i.e., the number of exosome particles) were obtained using an exosome protein standard curve provided in the ExoCET™ kit (System biosciences, USA) and calibrated to the Asys UVM 340 microplate reader (Biochrom).

### Nuclear DOX quantification

To evaluate the intracellular uptake of nanodrugs in the nucleus of cells, the uptake of free DOX, encapsulation-DOX, covalent conjugation-DOX and PEG coat-DOX by cancer (i.e., A549, MDA-MB-231) and normal (i.e., MRC-5, NHFB) cell lines were tested. The cells were seeded on glass coverslips (at a density of 3 × 10^4^ cells) and incubated for 24 hrs. The cells were then incubated with a medium containing the tested drug at the concentration of 200 ng/ml. After the designated treatment times (i.e., 0, 2, 6 and 12 hrs), the medium was removed and cells were washed two times using cold PBS. After each measurement, cells were mounted and visualized at the same setting using a confocal microscopy LSM700 (Carl Zeiss, Germany). All fluorescence intensities were analyzed using image J software (Java 1.6.0_24, NIH, USA).

### Measurement of mitochondrial membrane potential (Δψ_m_)

To measure the Δψ_m_, JC-1, a lipophilic cation-sensitive fluorescent probe for detecting Δψ_m,_ was used according to the manufacturer’s instructions (Molecular Probes). Cells were cultured on coverslips at a density of 2 × 10^4^ cells in 500 μl of culture medium overnight. Cells were then treated with the experimental drugs (i.e., DOX, encapsulation-DOX, PEG coat-DOX and covalent conjugation-DOX at concentrations of 200 ng/ml and placed in a CO_2_ incubator at 37 °C for 24 hrs. Cells were washed and incubated with 1 μM of JC-1 at 37 °C for 30 min in the absence of light. After removing the JC-1, images were captured by confocal microscopy (LSM700, Carl Zeiss, Germany) with both red and green channels. A total of 6 random, non-adjacent fields in each group were used for statistical analysis. Image J software was used to measure the average fluorescence intensity of the red and green fluorescence in each group. The Δψ_m_ level is represented by the JC-1 fluorescence ratio, calculated as the averaged fluorescence intensity ratio of red/green.

### *In vitro* detection of ROS

For *in vitro* ROS detection, cells were pre-treated with the experimental drugs (i.e., DOX, encapsulation-DOX, PEG coat-DOX and covalent conjugation-DOX at concentrations of 200 ng/ml for 24 hrs. The ROS dihydroethidium bromide (DHE) probe was then added to cells at a concentration of 400 nM and incubated at 37 °C for 10 min. In the presence of ROS, DHE rapidly oxidizes and forms a highly fluorescent product. The ROS signal from various treatments was obtained at the same setting using confocal microscopy LSM700 (Carl Zeiss, Germany). Fluorescence quantification was performed using Image J software to measure the red fluorescence integrated intensity.

### Measurement of extracellular pH and the acidification rate on intracellular pH (pH_*i*_)

Intracellular pH (pH_*i*_) was measured using the classical pH calibration method, or the nigericin and high-K^+^ method, to monitor pH regulation in single glomerular mesangial cells^52^. I. Acid extrusion in the absence or presence of HCO_3_^−^ results in the pH of the intracellular components to equilibrate with the extracellular pH^53^. Thus, the difference in absolute pH between different compartments in diverse cell types is not mimicked by the pH calibration method as mentioned^54^. To determine the pH_*i*_ modulation in each cell types, the slope of acidification (∆pH/sec) in the presence of CO_2_/HCO_3_^−^. pH_*i*_ was measured with 2′,7′-Bis-(2-Carboxyethyl)-5-(and-6)-carboxyfluorescein, acetoxymethyl ester (BCECF-AM, Teflabs) at dual excitation wavelengths of 495 nm and 440 nm. BCECF fluorescence was read at emission wavelengths greater than 530 nm. Cells attached onto coverslips were loaded in the chamber with BCECF in the presence of 0.05% Pluronic F-127 (Invitrogen) and incubated for 15 min in physiological salt solution (i.e., PSS, 140 mM NaCl, 5 mM KCl, 1 mM MgCl_2_, 1 mM CaCl_2_, 10 mM Hepes, and 10 mM glucose, pH 7.4) at RT with 6 μM BCECF-AM. After stabilizing the fluorescence, the cells were perfused with PSS for at least 10 min before measuring pH_i_ at 37 °C. To measure the acidification rate of cytosol, cells were perfused by CO_2_-saturated HCO_3_^–^-buffered media. The emitted fluorescence was monitored with a CCD camera (Photometrics, Tucson, AZ) attached to an inverted microscope (Olympus, Japan) and analyzed with a MetaFluor system (Molecular Devices, PA). Fluorescence images were obtained at 1 sec intervals and the background fluorescence was subtracted from the raw background signals at each wavelength. The acidification rate, given as ∆pH_*i*_/sec, was determined from the slopes, the derivatives of the first 20–35 sec of pH_i_ decreases in CO_2_-saturated HCO_3_^−^-buffered media. The extracellular pH was measured directly with a pH meter from cell media.

### Cell viability and apoptosis

Cancer and normal cells (seeded at 5 × 10^3^ cells/well in 96-well plates) were treated with various concentrations of the experimental nanodrugs and incubated at 37 °C for 48 hrs. Cell viability was determined using the 3-(4,5-dimethylthiazolyl-2)2, 5-diphenyl tetrazolium bromide assay (MTT, Amresco). After 48 hrs of treatment, 100 μl of MTT (1 mg/ml) was added to each sample well and the samples were incubated for 2 hrs. DMSO was added to dissolve the formazan crystals. The absorbance of each sample was calculated relative to that of the control and expressed as a percentage increase. In order to investigate the effect of nano-conjugation on cell apoptosis, cancer and normal cells were seeded in 6-well plates (at a density of 5 × 10^5^ cells/well) and treated with the nanodrugs (i.e., DOX, encapsulation-DOX, covalent conjugation-DOX and PEG coat-DOX) for 24 hrs. Cells without treatment were used as a control. The percentage of apoptosis was determined by staining with annexin V. After 24 hrs of incubation, cells were removed and prepared in accordance with the protocol of Annexin V-Pacific Blue™ conjugate (A35122, Invitrogen, Carlsbad, CA, USA). In short, 5 μl of Annexin V- Pacific Blue™ conjugate was added to each sample and incubated for 15 min in the absence of light at room temperature. Cells were then resuspended in 400 μl of PBS (supplemented with 1% FBS) and analyzed by BD LSR II flow cytometer (Becton Dickinson, Germany) at 410 nm excitation and with a 455 nm bandpass filter for Pacific blue detection.

### Statistical analysis

The statistical differences between mean values obtained from two sample groups were analyzed with the Student’s t-test. The differences were considered significant if the P value was less than or equal to 0.05. The statistical differences between several sample types were analyzed with ANOVA followed by the Newman-Keuls’ multiple comparison test. Asterisks (*, **, and ***) indicated the significance of p values less than 0.05, 0.01, and 0.001, respectively.

## Electronic supplementary material

Supplementary Information
