# Supplementary material for: Covalent, Non-Covalent, Encapsulated Nanodrug Regulate the Fate of Intra- and Extracellular Trafficking: Impact on Cancer and Normal Cells
Source: Sci Rep. 2017 Jul 25;7:6454. doi: 10.1038/s41598-017-06796-7 (PMC5526881; doi:10.1038/s41598-017-06796-7)
Supplement: Supplementary file 1 — Supplementary Information [file 41598_2017_6796_MOESM1_ESM.pdf]

Supplementary information for

Covalent, Non-Covalent, Encapsulated Nanodrug Regulate the Fate of Intra- and Extracellular Trafficking: Impact on Cancer and Normal Cells

Sang-Woo Kim[1,*], Yeon Kyung Lee[1,*], Sang-Hyun Kim[2,*], Jun-Young Park[1], Dong Un Lee[1], Jungil Choi[3], Jeong Hee Hong[1,4,*], Sanghyo Kim[5,†] & Dongwoo Khang[1,4,†]

[1]*Lee Gil Ya Cancer and Diabetes Institute, Gachon University, Incheon 21999, South Korea*

[2]*Department of Pharmacology, Kyungpook National University, Daegu 41566, South Korea*

[3]*Gyeongnam Department of Environment Toxicology and Chemistry, Korea Institutes of Toxicology, Jinju 52834, Republic of Korea*

[4]*Department of Physiology, Gachon University, Incheon 21999, South Korea*

[5]*Department of Bionanotechnology, Gachon University, Seongnam 13120, South Korea*

[*]These authors contributed equally to this work.

[†]Correspondence and request for materials should be addressed to

D.K. (email: dkhang@gachon.ac.kr) or S.K. (email: samkim@gachon.ac.kr).

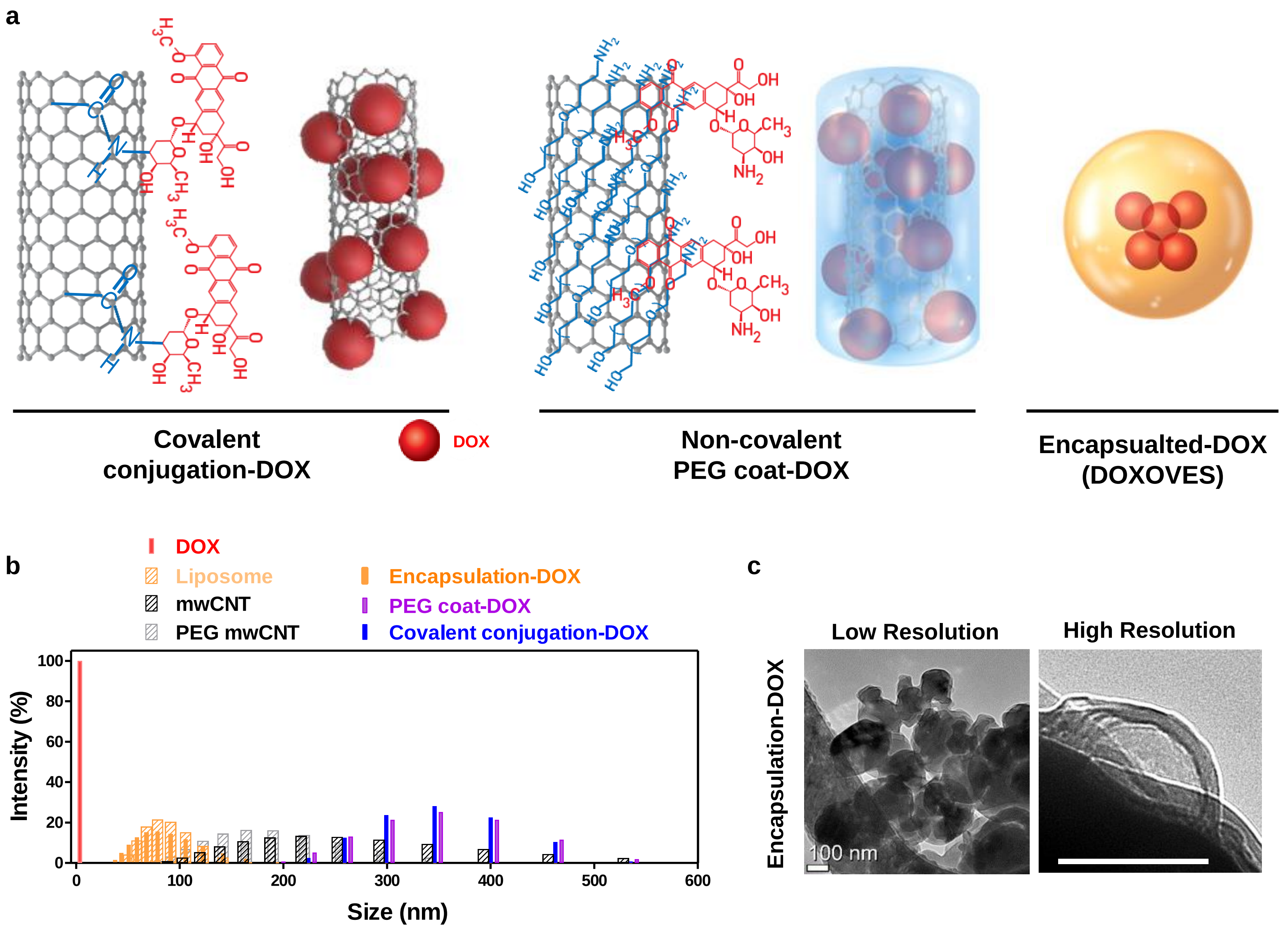
a
Covalent conjugation-DOX
DOX
Non-covalent PEG coat-DOX
Encapsualted-DOX (DOXOVES)
b
DOX
Liposome
mwCNT
PEG mwCNT
Encapsulation-DOX
PEG coat-DOX
Covalent conjugation-DOX
Intensity (%)
Size (nm)
c
Low Resolution
High Resolution
Encapsulation-DOX
100 nm

Figure S1. Histogram of particle size distribution and Cryo-TEM image of PEG coat mwCNT. (a) Chemical illustration of covalent conjugation-DOX, PEG coat-DOX and encapsulated DOX (DOXOVES) **(b)** Particle sizes of tested samples in PBS (pH 7.2) solution. Histogram represented the size distribution of nanodrugs ranges from 80 nm to 350 nm. **(c)** High and low resolution of Cryo-TEM images showed morphology of encapsulated DOX (DOXOVES) in PBS solution. Scale bar is 100 nm (right).

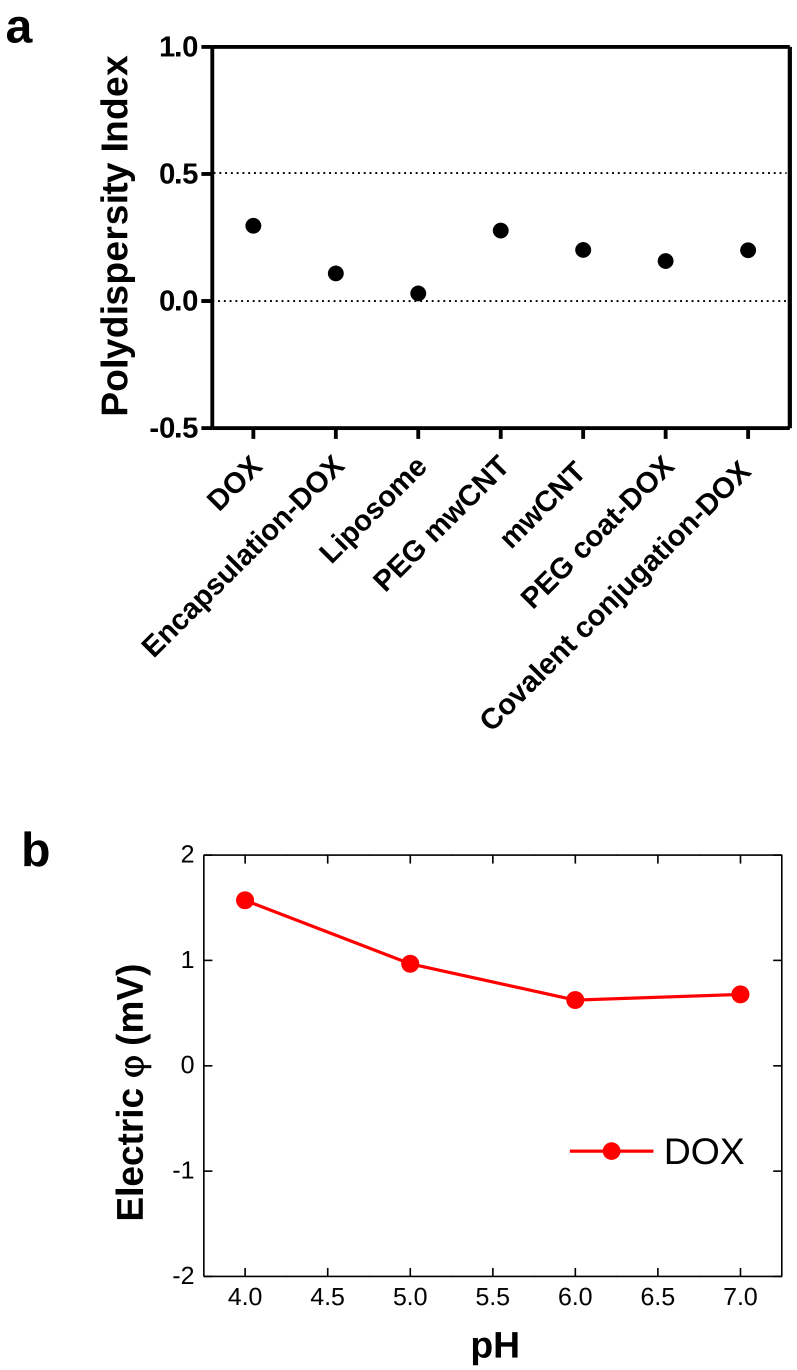

Figure S2. PDI values and electric potential changes of DOX by the variation of pH. (a) PDI values were less than 0.5 for all tested samples. **(b)** The electric potential of DOX was measured by changing pH from 4 to 7. Net potential change (pH 4~7) in DOX was negligible.

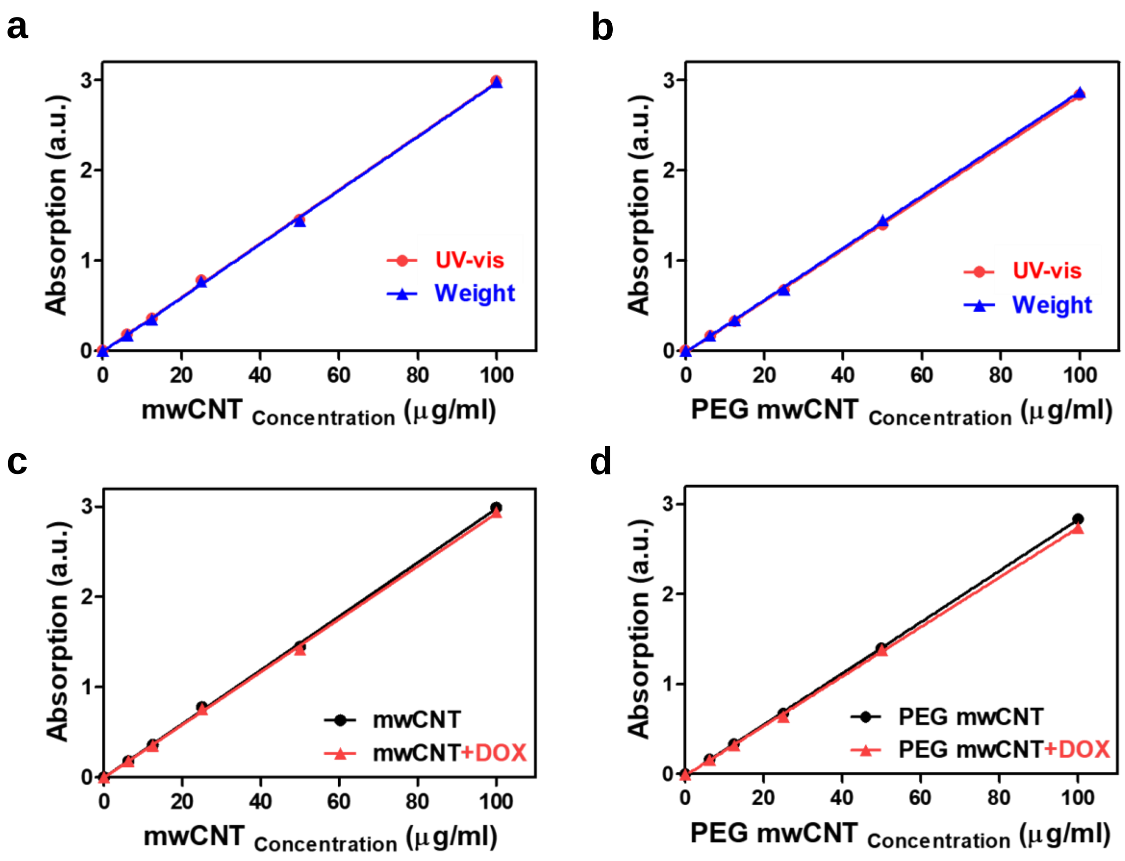

Figure S3. Comparison of UV-vis and weight standard curves. (a and b) Comparison between UV-vis and weight standard curves of **(a)** mwCNT and **(b)** PEG coated mwCNT. UV-vis and weight of carboxylated CNT and PEG coated CNT were directly measured and the reciprocal coincidence of weight was compared to each other. Standard curves convinced no differences between UV-vis and balance. **(c and d)** Interferences of adding DOX for **(c)** carboxylated CNT and negligible interference of **(d)** PEG coated CNT by adding DOX was confirmed by UV-vis analysis.

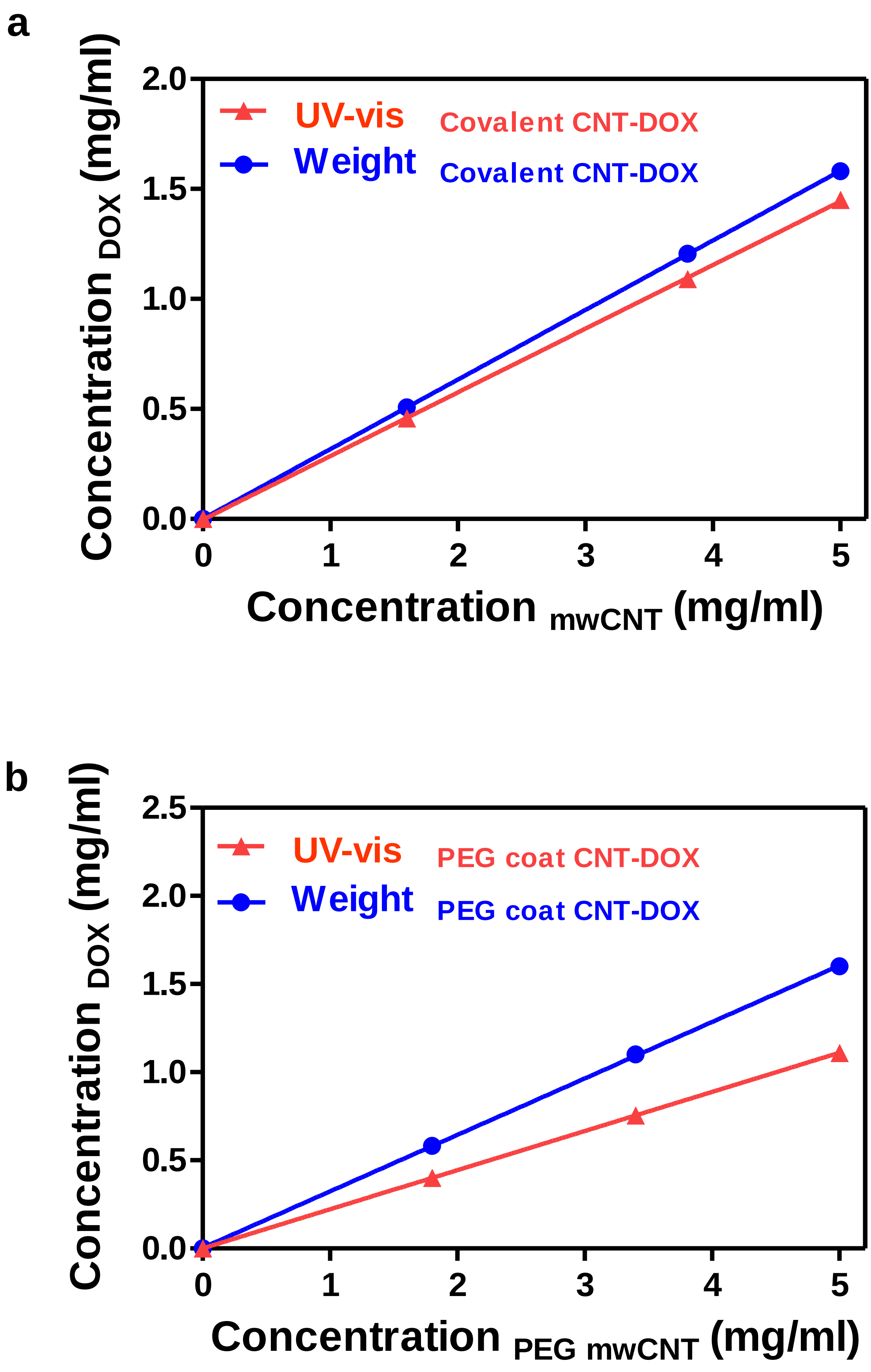
a
UV-vis Covalent CNT-DOX
Weight Covalent CNT-DOX
Concentration DOX (mg/ml)
Concentration mwCNT (mg/ml)
0.0
0.5
1.0
1.5
2.0
0
1
2
3
4
5
b
UV-vis PEG coat CNT-DOX
Weight PEG coat CNT-DOX
Concentration DOX (mg/ml)
Concentration PEG mwCNT (mg/ml)
0.0
0.5
1.0
1.5
2.0
2.5
0
1
2
3
4
5

Figure S4. Comparison of UV-vis and weight standard curves of covalently conjugated DOX and PEG coated DOX. (a and b) Differences between UV-vis and weight standard curves of **(a)** covalently conjugated DOX **(b)** PEG coated DOX (CNT was used as base material). Data exhibited the discrepancy between the UV-vis and weight standard curves of tested nanodrugs. Based on these standard curves, corrected weights of nanodrugs were used in this study.

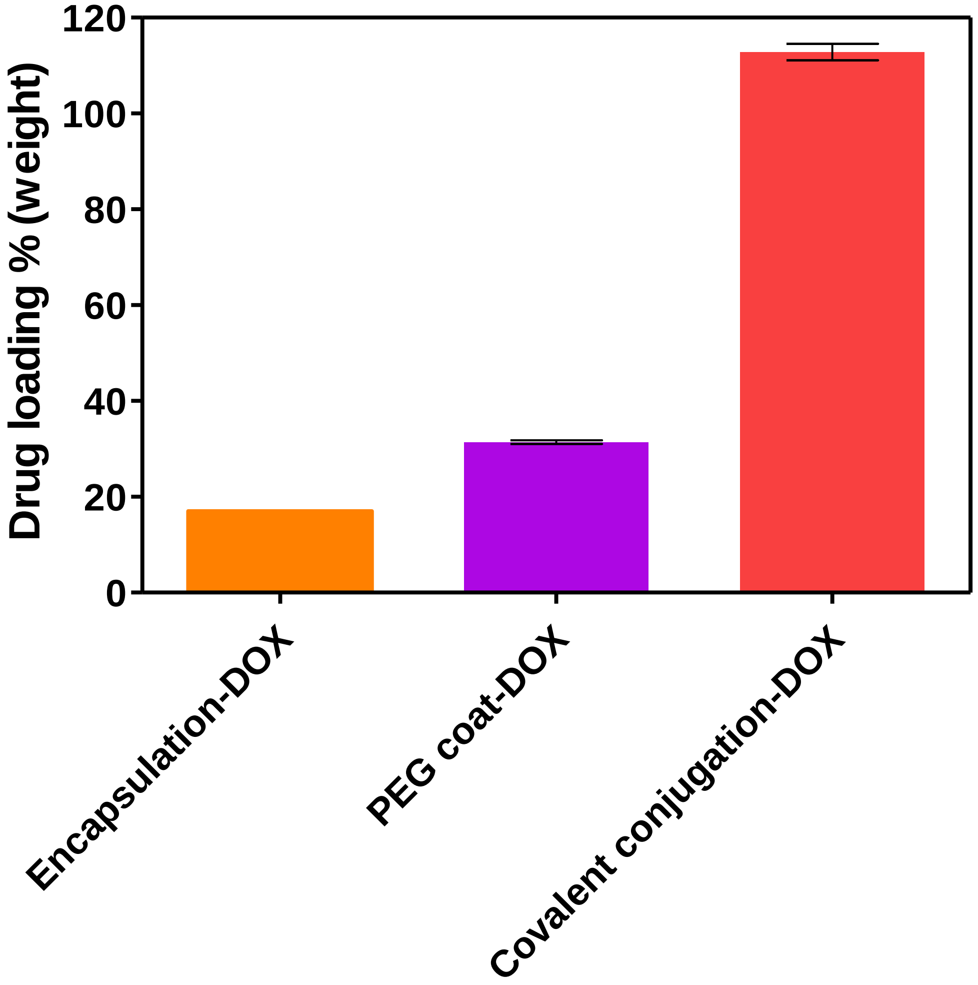

Figure S5. DOX loading (Weight %) of nanodrugs. The weight ratio of loaded nanodrug (i.e., encapsulation-DOX, PEG coat-DOX and covalent conjugation-DOX) compared to the weight of base materials (i.e., liposome, PEG coat CNT and carboxylated CNT). The mean weight ratios of loaded drugs were 112 %, 31.4 % and 17.0 % for covalent conjugation-DOX, PEG coated-DOX and encapsulation-DOX, respectively. Data represent mean ± SEM (n=5).

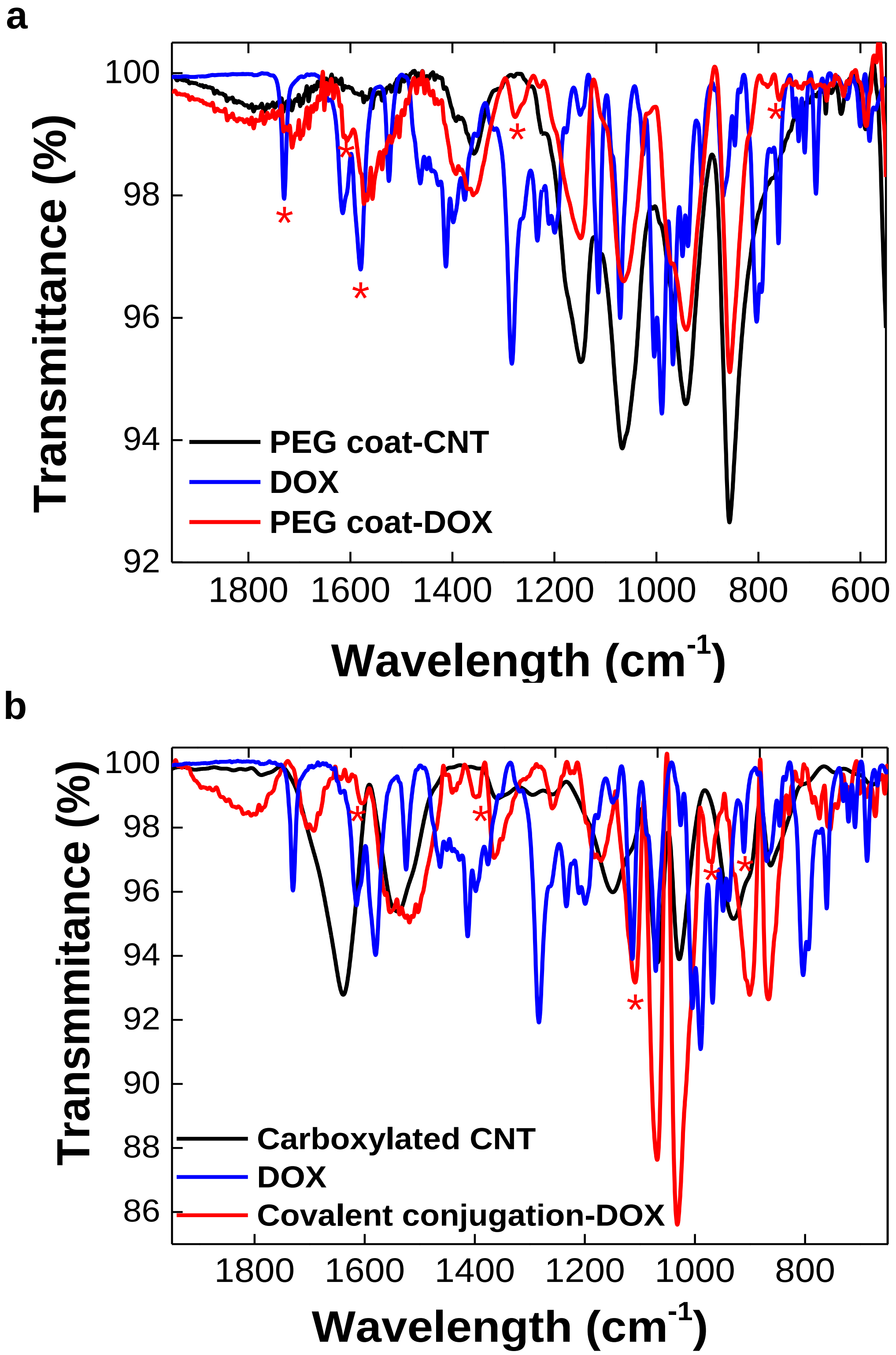
a
Transmittance (%)
100
98
96
94
92
1800
1600
1400
1200
1000
800
600
Wavelength (cm-1)
PEG coat-CNT
DOX
PEG coat-DOX
b
Transmmitance (%)
100
98
96
94
92
90
88
86
1800
1600
1400
1200
1000
800
Wavelength (cm-1)
Carboxylated CNT
DOX
Covalent conjugation-DOX

Figure S6. FTIR analysis of nanodrugs. (a-c) Loaded drugs (DOX) on nanomaterials were identified by FTIR. FTIR spectra confirm a coincidence of several IR peaks (red symbol) between the free drug and conjugated drugs. FTIR spectra shows that major molecular vibration peaks of **(a)** PEG coated-DOX and **(b)** covalent conjugation-DOX (red color) were consistent with peaks from DOX and, thus, supported the evidence of drug conjugation on base nanomaterials.

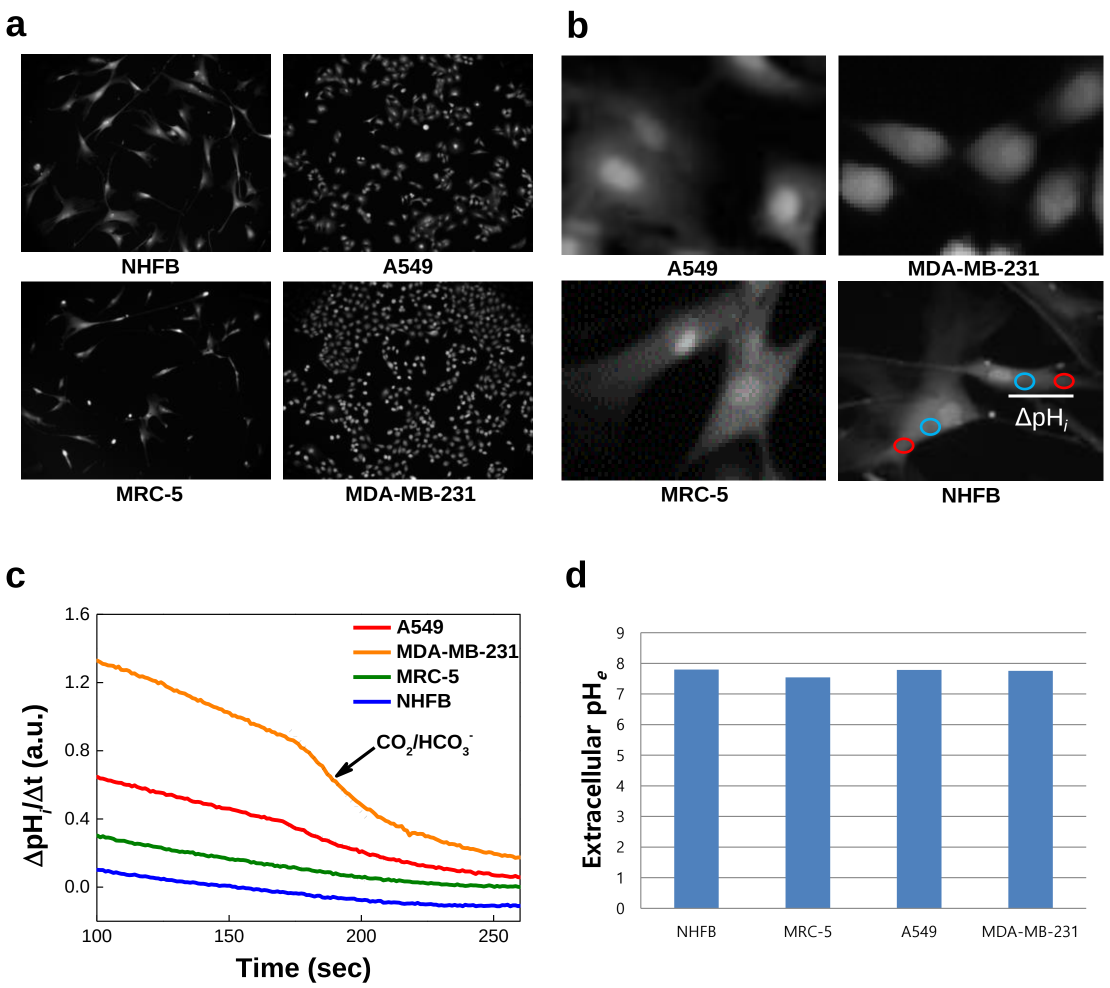

Figure S7. Intra- and extra-cellular pH of cancer and normal cells. (a) Fluorescence images (far-field view) showed BCECF (pH-indicator) stained cancer (A549 and MDA-MB-231) and normal (MRC-5 and NHFB) cells. Far-field view of cancer and normal cells. **(b)** Magnified images represented that ΔpH_i was analyzed by measuring pH_i between near extracellular membrane (blue circle) and near nucleus region (red circle) of cytosol in tested cells. **(c)** Sharp slope of acidification (ΔpH/sec) were observed in cancer cells, whereas flat slope of acidification were found in normal cells. These results represent that significant changes of pH_i were only detected in cancer cells. **(d)** No significant changes in extracellular pH in media were identified for tested cells (confined only *in vitro* condition).

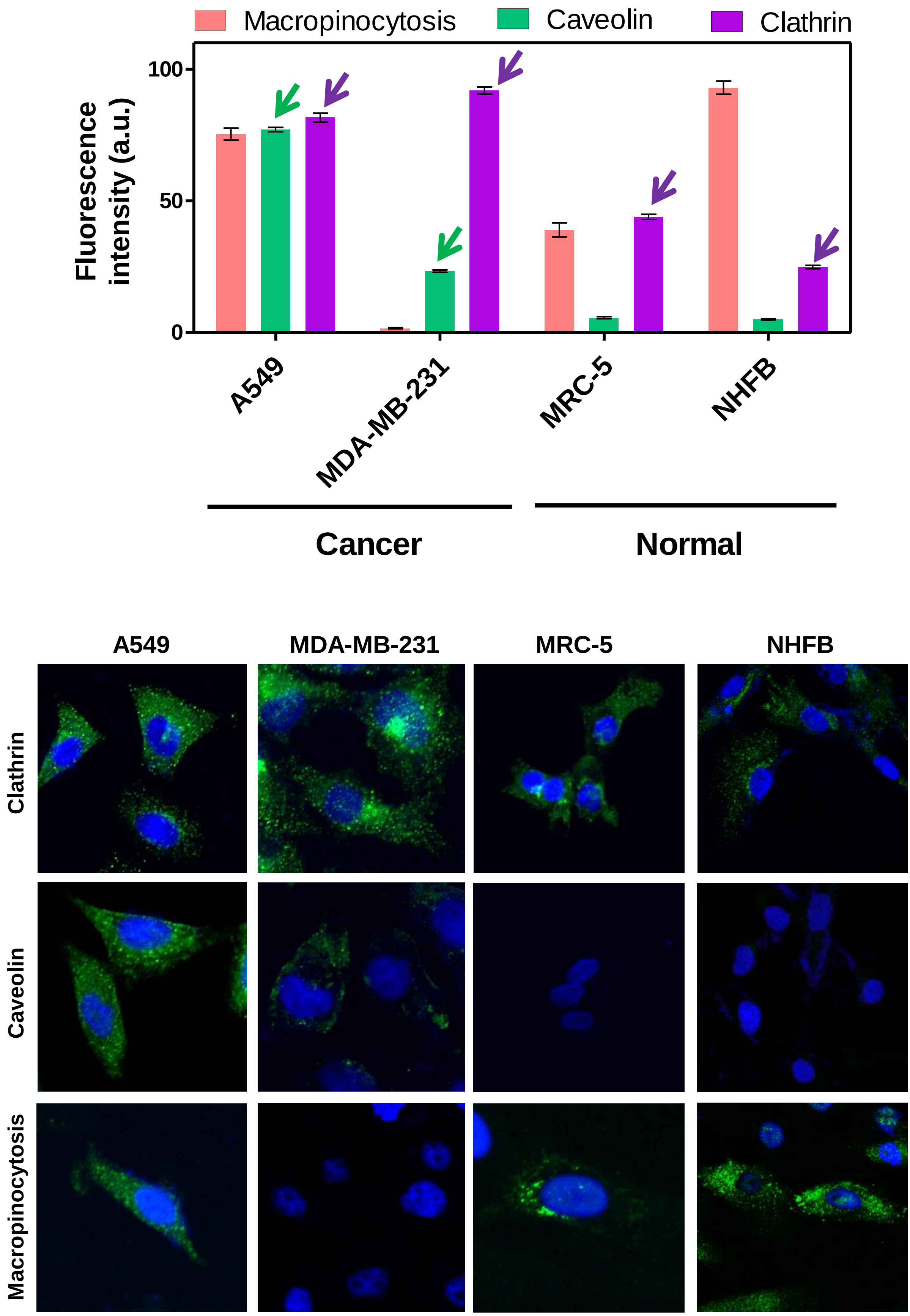

Macropinocytosis
Caveolin
Clathrin
Fluorescence intensity (a.u.)
100
50
0
A549
MDA-MB-231
MRC-5
NHFB
Cancer
Normal
A549
MDA-MB-231
MRC-5
NHFB
Clathrin
Caveolin
Macropinocytosis

Figure S8. Intrinsic expression of ATP assisted uptake markers (e.g., macropinocytosis, caveolin and clathrin) in cancer and normal cells. Fluorescence intensity (upper) was evaluated and the expression of each active uptake marker was visualized, where green and blue correspond to the uptake marker-specific antibody and DAPI nucleus staining, respectively, using fluorescent confocal microscopy. All tested cells (i.e., both cancer and normal) exhibited positive clathrin uptake. Normal cells tested positive for macropinocytosis, but negligible for caveolin markers.

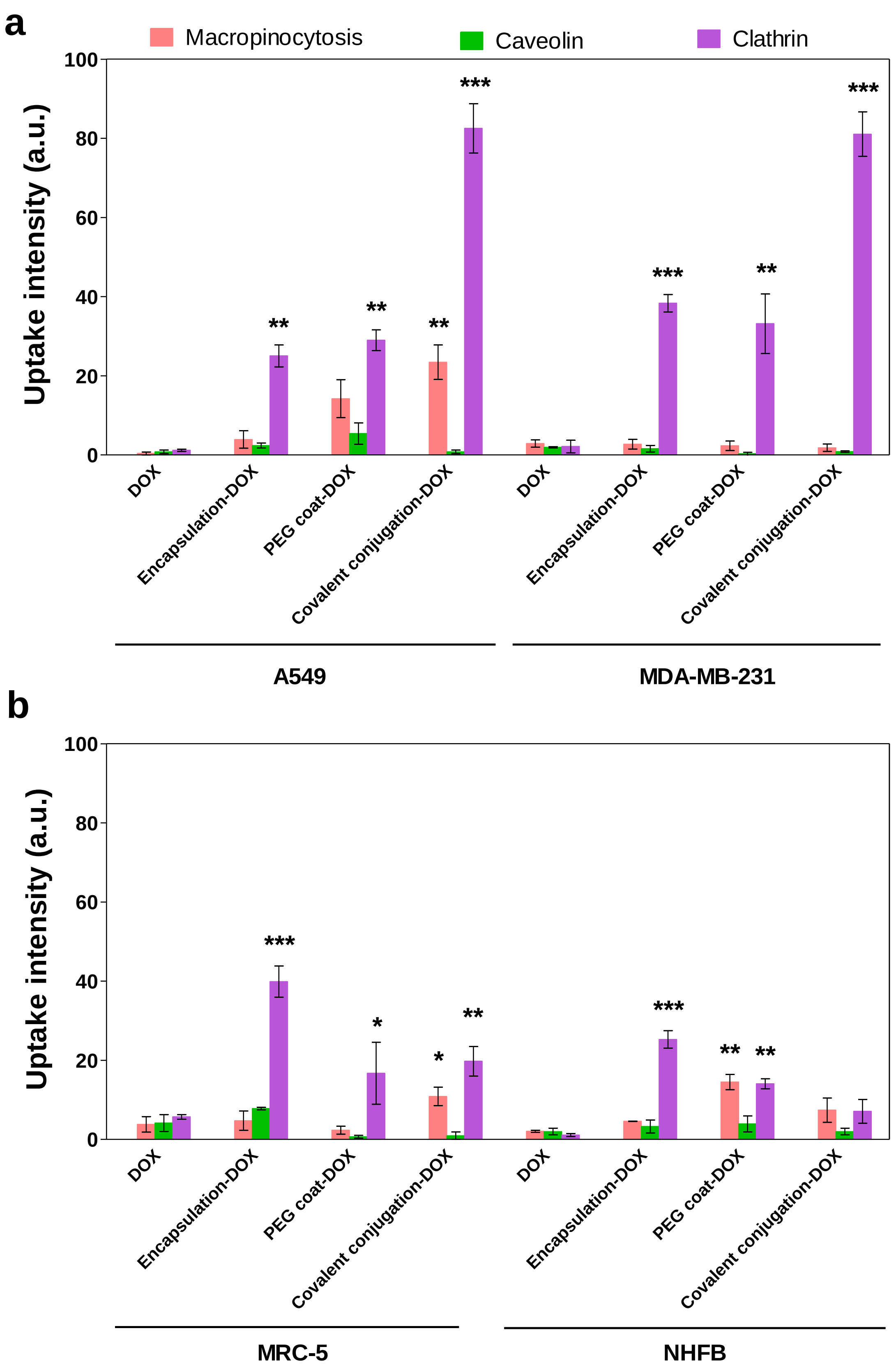

a
Macropinocytosis
Caveolin
Clathrin
Uptake intensity (a.u.)
100
80
60
40
20
0
***
**
**
**
***
**
***
DOX
Encapsulation-DOX
PEG coat-DOX
Covalent conjugation-DOX
A549
MDA-MB-231
b
Uptake intensity (a.u.)
***
*
*
**
***
**
**
MRC-5
NHFB

Figure S9. Uptake pathways of nanodrugs. The relative FACS uptake expression of various nanodrugs in **(a)** cancer cells (A549 and MDA-MB-231) and **(b)** normal cells (MRC-5 and NHFB). Endocytosis inhibitors (e.g., macropinocytosis; EIPA, caveolin; GEN, clathrin; CPZ) were used to analyze uptake response. Notably, greater clathrin uptake was observed in covalently conjugated nanodrugs, as compared with other conjugation types. All data represent the mean ± SEM ($n = 5$). *, ** and *** correspond to $p < 0.05$, $p < 0.01$ and $p < 0.001$, respectively, as compared with untreated controls (i.e., black bar).

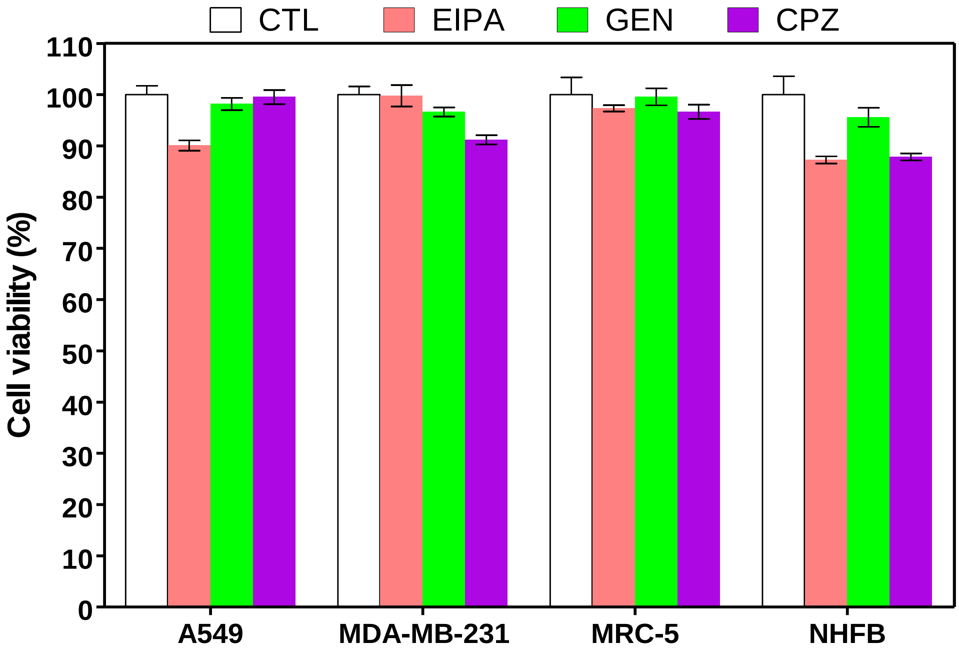

Figure S10. Toxicity evaluations of uptake inhibitors on cancer and normal cells by MTT assay following 3 hrs incubation. Toxicity of uptake inhibitors (i.e., EIPA, genistein (GEN), and chlorpromazine (CPZ)) was evaluated in a given concentration on cancer (A549 and MDA-MB-231) and normal cells (MRC-5 and NHFB) by MTT assay. Inhibitor concentration was 25 μM, 200 μM and 20 μM for EIPA, GEN and CPZ, respectively. At least, 80-90 % of cell viabilities were maintained for all tested cells after 3 hrs.

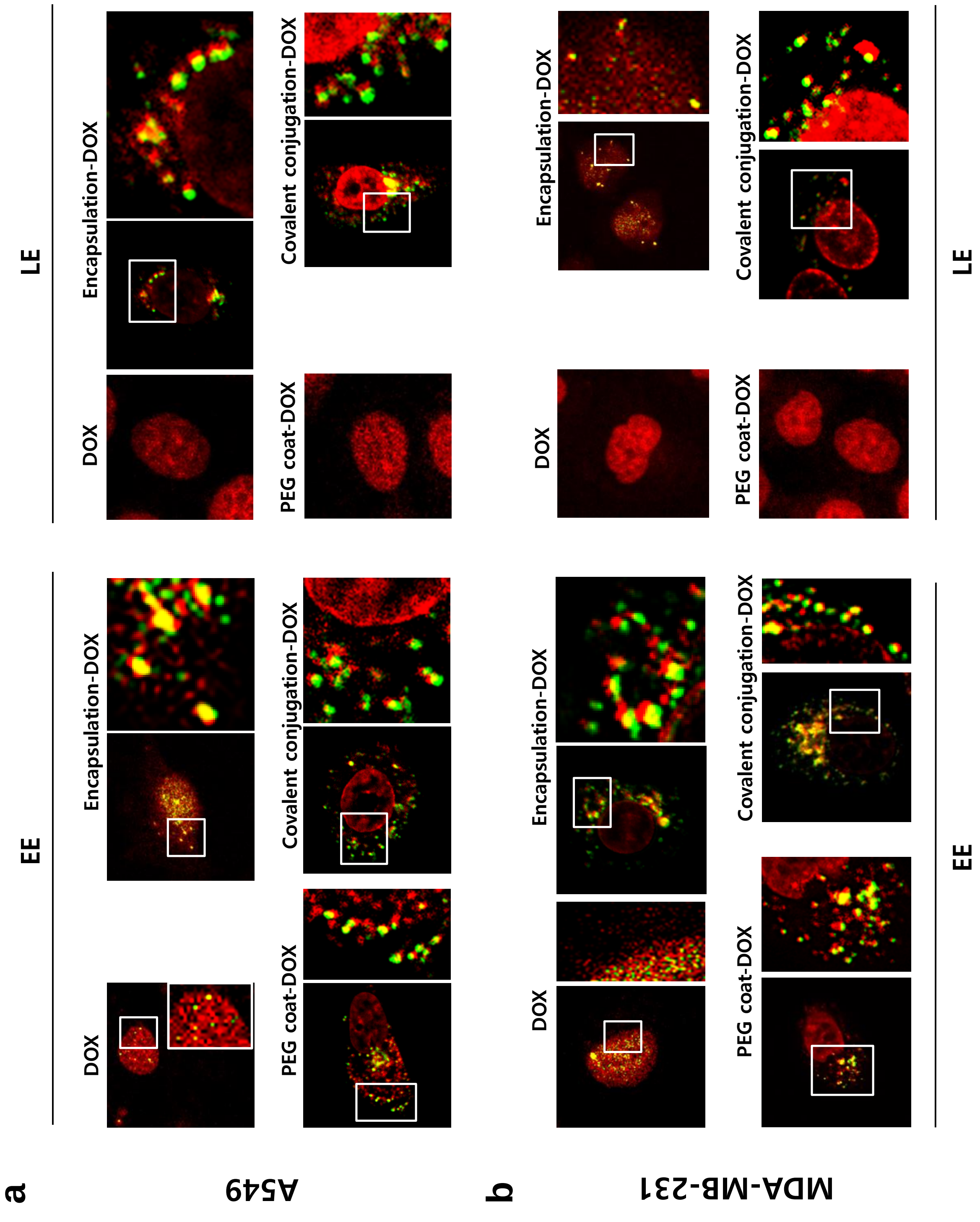

Figure S11. Co-localization analysis of nanodrugs with endocytic vesicles (EE or LE). Confocal images show co-localized boundaries between nanodrugs (i.e., encapsulation, PEG coat and covalent conjugation; (red)) with EE (green, anti-EEA1) or LE (green, anti-mannose 6-phosphate receptor (M6PR)) in **(a)** A549 and **(b)** MDA-

MB-231 cells. Yellow color designate co-localized region between nanodrugs and vesicles (EE (at 6 hrs) and LE (at 12 hrs)).

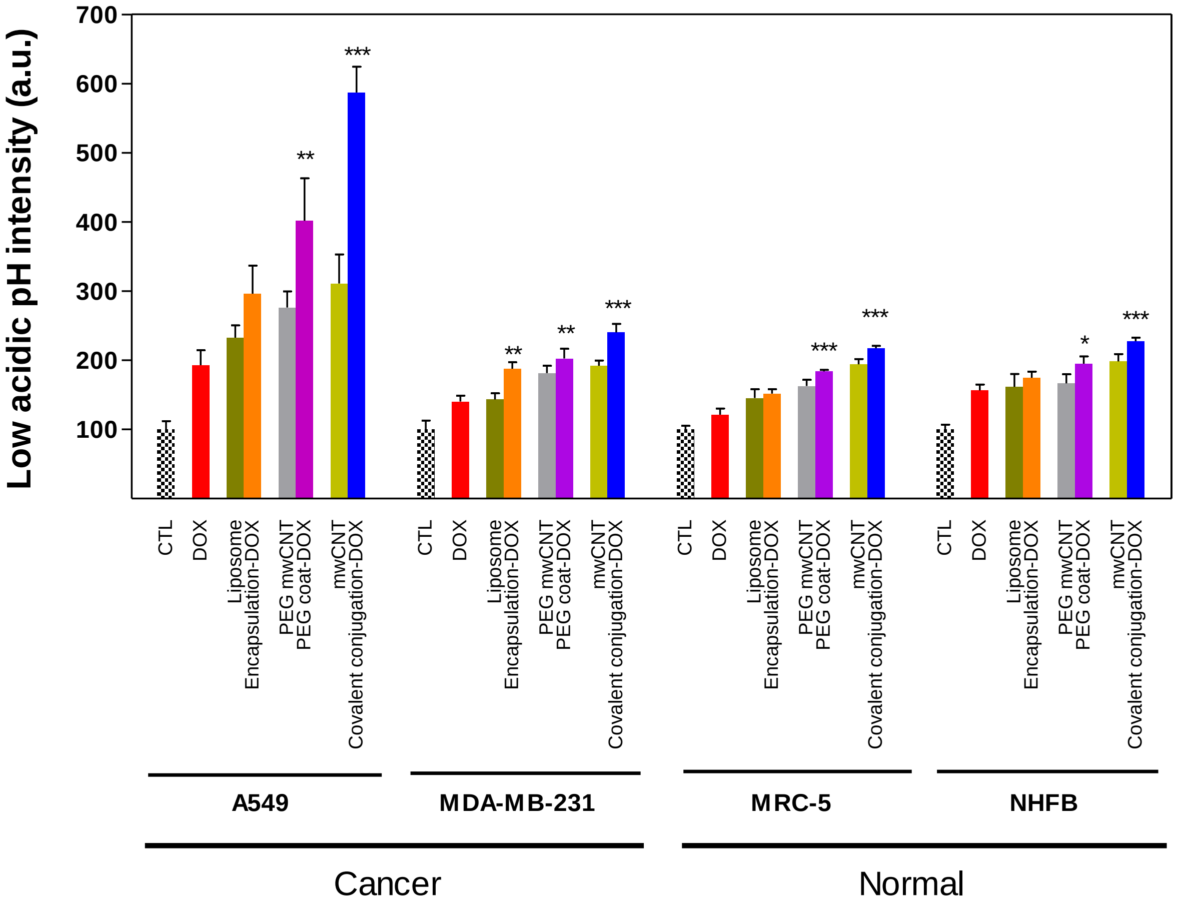

Figure S12. Low acidic pH intensity of nanodrugs in cancer and normal cells. Low acidic pH organelle intensity was analyzed after 24 hrs in cancer and normal cells using lysotracker. Low acidic pH organelle intensity increased significantly on PEG coat-DOX and covalent conjugation-DOX compared to other drugs. Specifically, covalently conjugated DOX elicited the highest low acidic pH intensity in cancer cells and normal cells. All data represent mean ± SEM (n=10). *$p < 0.05$, **$p < 0.01$, and ***$p < 0.001$ *vs.* DOX.

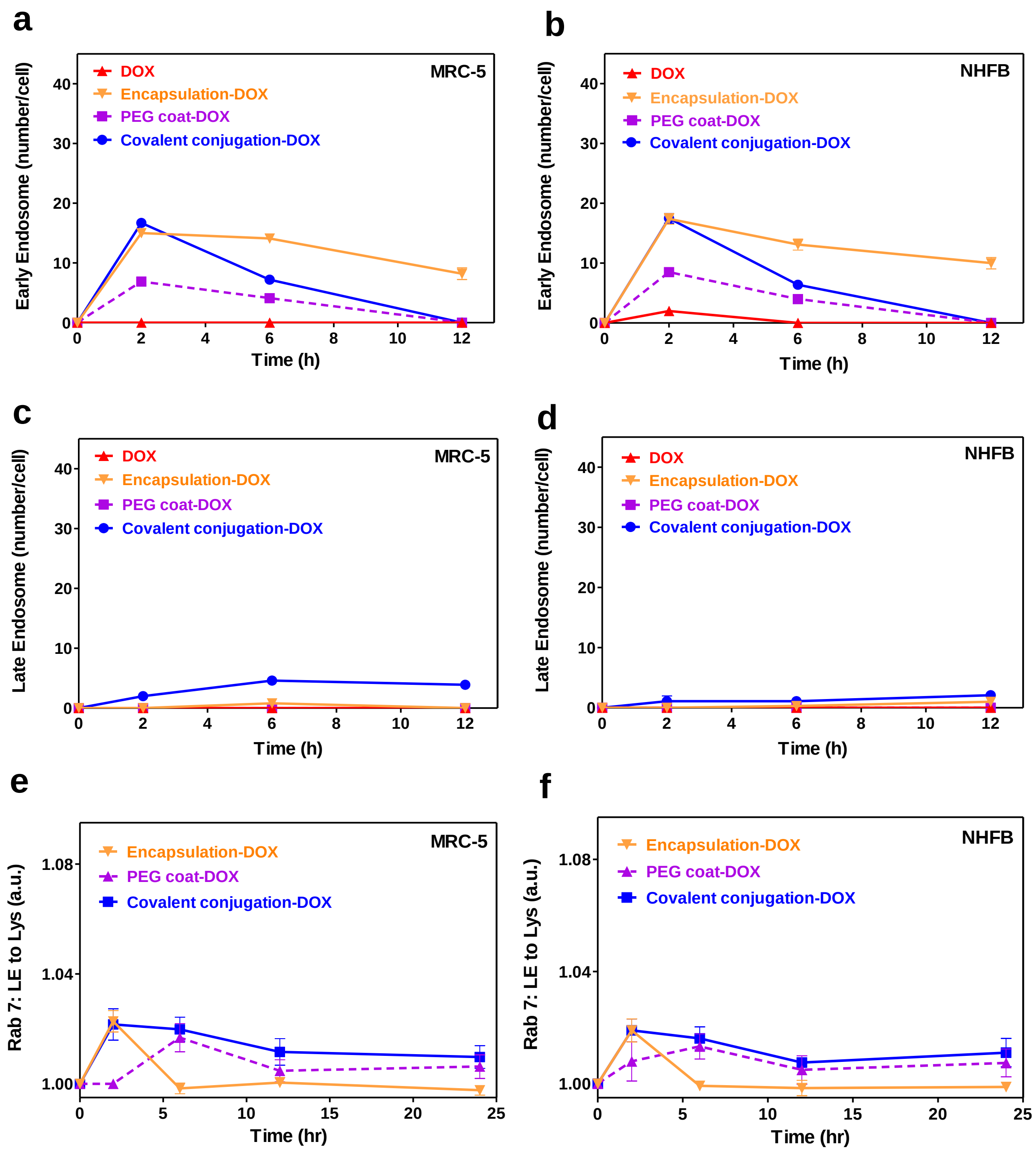

Figure S13. Intracellular trafficking (EE, LE and Lys) of nanodrugs in normal cells. (a-d) Quantification of time-dependent co-localization from confocal microscopy images. Time-dependent co-localization of each nanodrug and EE for **(a)** A549 and **(b)** MDA-MB-231 cells. Time-dependent co-localization of each nanodrug and LE in **(c)** A549 and **(d)** MDA-MB-231 cells. Most of tested drugs developed from

EE to LE except DOX treated cells. The formation rate and number of LE vesicles in all tested samples was significantly lower than that of cancer cells. **(e-f)** Rab7 expression profile (indicative of LE to Lys). Rab7 expressions for tested nanodrugs were non-significant for both **(e)** MRC-5 and **(f)** NHFB cells (until approximately 24 hrs). All data represents the mean ± SEM (n=10).

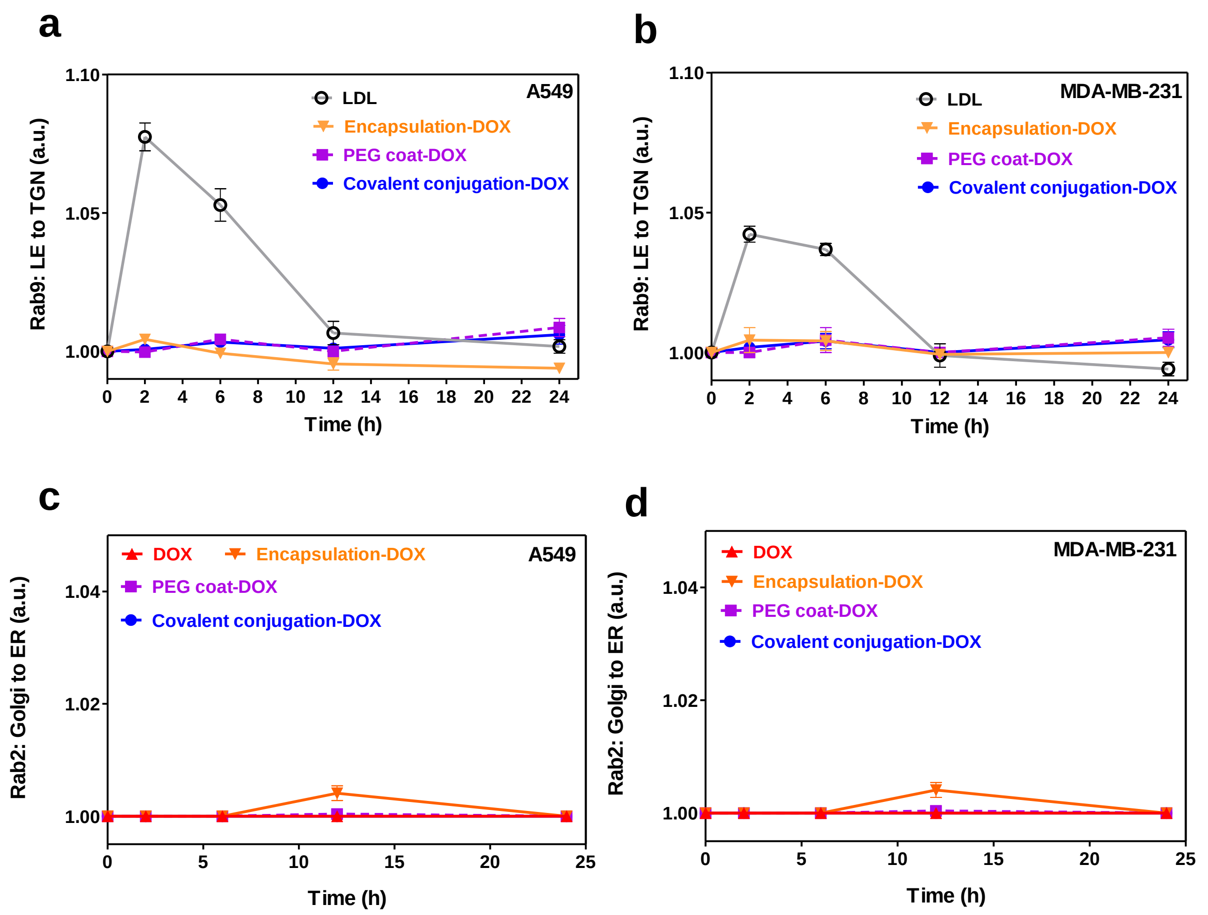

Figure S14. Intracellular trafficking (LE to TGN and Golgi to ER) of nanodrugs in cancer cells. Time dependent Rab9 expression (indicative of LE to TGN) in **(a)** A549 and **(b)** MDA-MB-231 cells. LDL (grey) was used as a positive marker for Rab9. All types of nanodrugs showed no transportation from the LE to the TGN. Time dependent Rab2 expression (indicative of Golgi to ER) in **(c)** A549 and **(d)** MDA-MB-231 showed no drug transport from Golgi to ER.

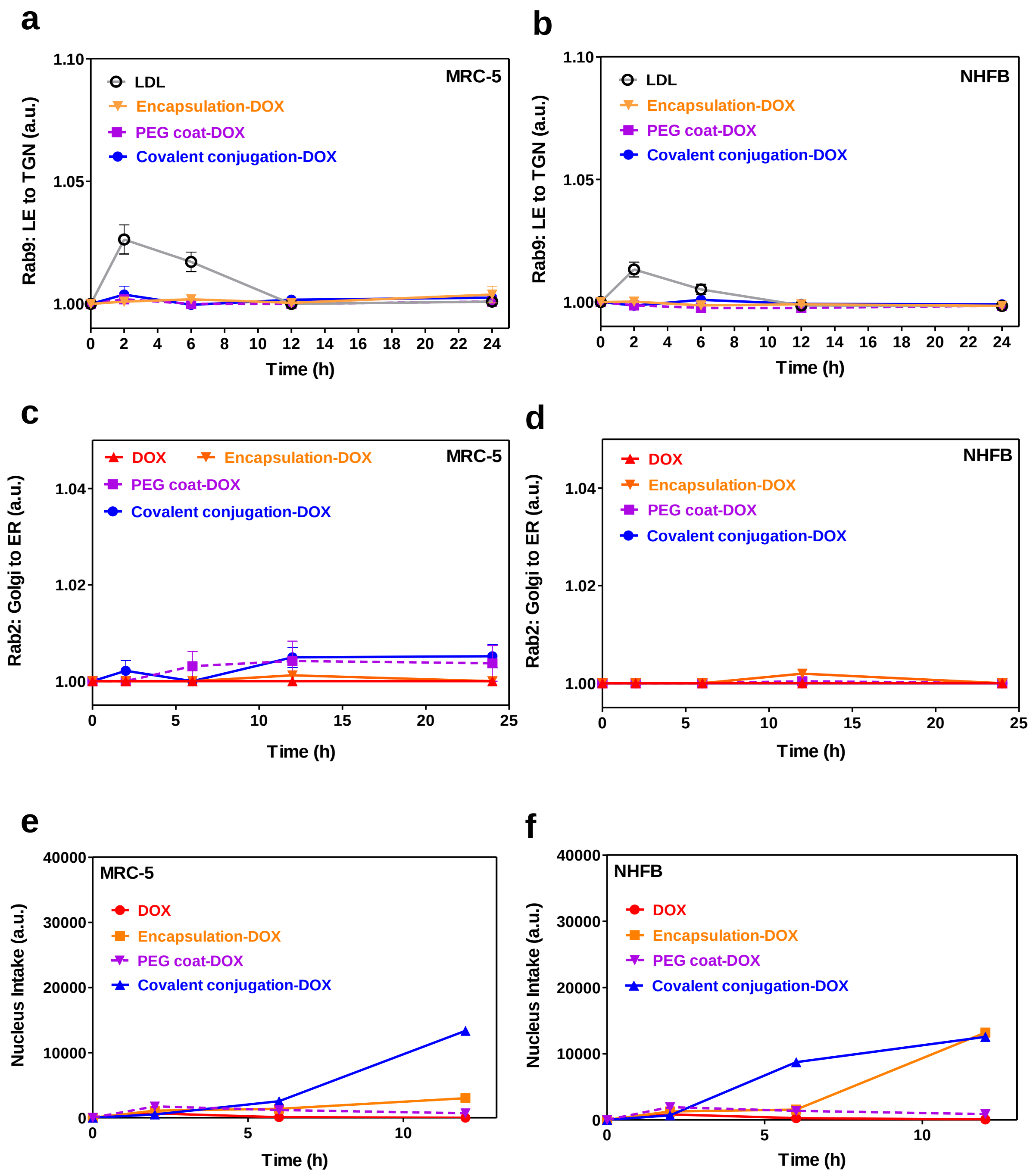

Figure S15. Intracellular trafficking (LE to TGN, Golgi to ER and nucleus intake) of nanodrugs in normal cells. Time-dependent Rab9 expression (indicative of LE to trans-Golgi network (TGN)) in **(a)** MRC-5 and **(b)** NHFB cells. LDL (low-density lipoproteins; grey) was used as a positive marker for Rab9. All types of nanodrugs showed no transportation from the LE to the TGN. Time-dependent Rab2 expression

(indicative of Golgi to ER) in **(c)** MRC-5 and **(d)** NHFB showed no drug transport from Golgi to ER. Time-dependent fluorescence intensity corresponding to DOX intake within the nucleus of **(e)** MRC-5 and **(f)** NHFB cells by the various types of nano-conjugations. Only covalent conjugation induced DOX fluorescence in the nuclei of MRC-5. And both covalent conjugation and liposomal encapsulation induced DOX fluorescence in the nuclei of NHFB after 10 hrs.

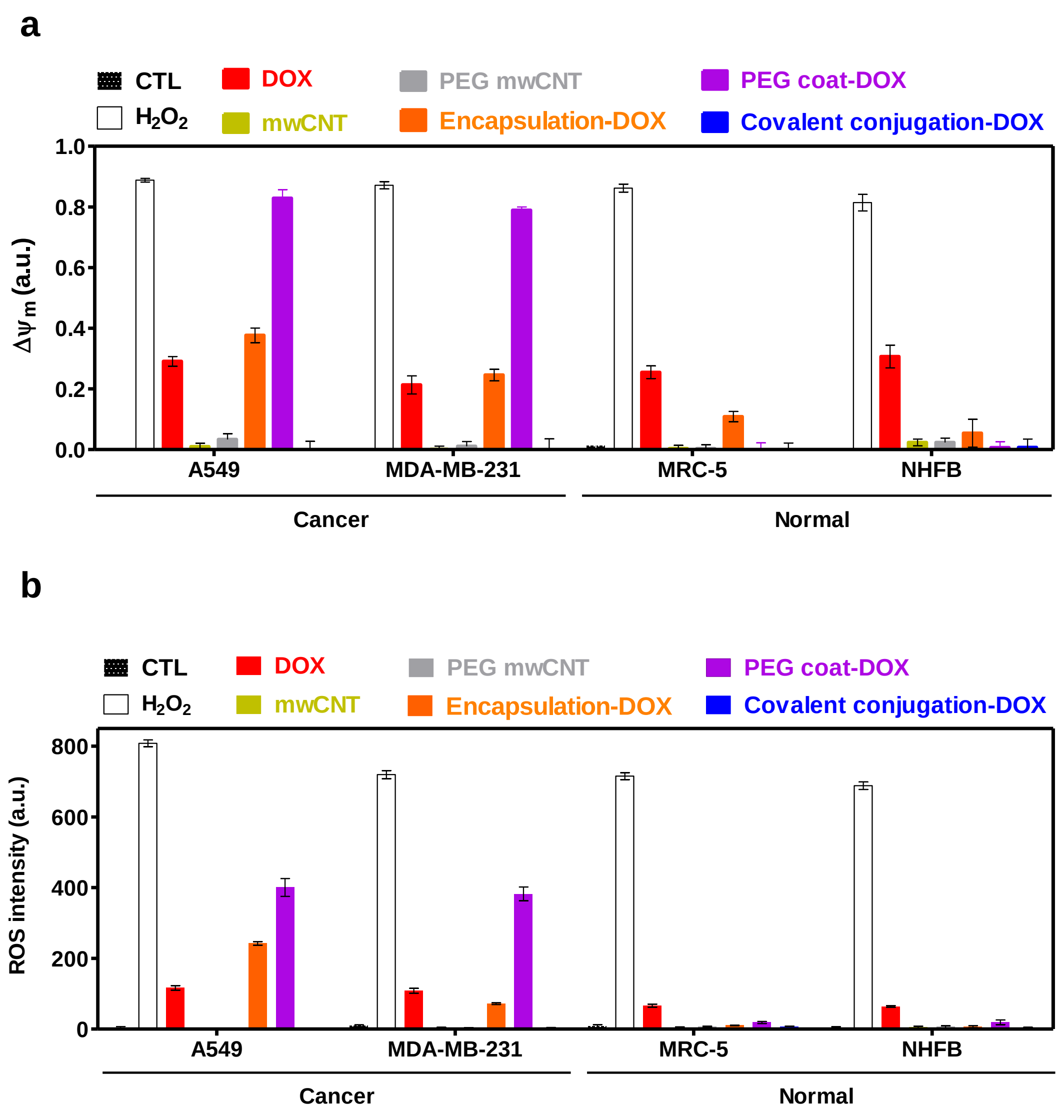

Figure S16. Changes in mitochondrial membrane potential ($\Delta\Psi_m$) and fluorescent ROS intensity (DHE) with nanodrug treatment for 24 hrs. (a) The JC-1 fluorescence ratio was calculated by the optical fluorescence density ratio of red/green in each group. PEG coated conjugations of DOX significantly influenced mitochondrial membrane potential in cancer cells (i.e., both A549 and MDA-MB-231), whereas normal cells (i.e., MRC-5 and NHFB) did not show any notable changes in membrane potential. **(b)** Fluorescence quantification was obtained by measuring DOX fluorescence intensity from confocal images (Fig. 4b). All data represent the mean ± SEM (n = 5).

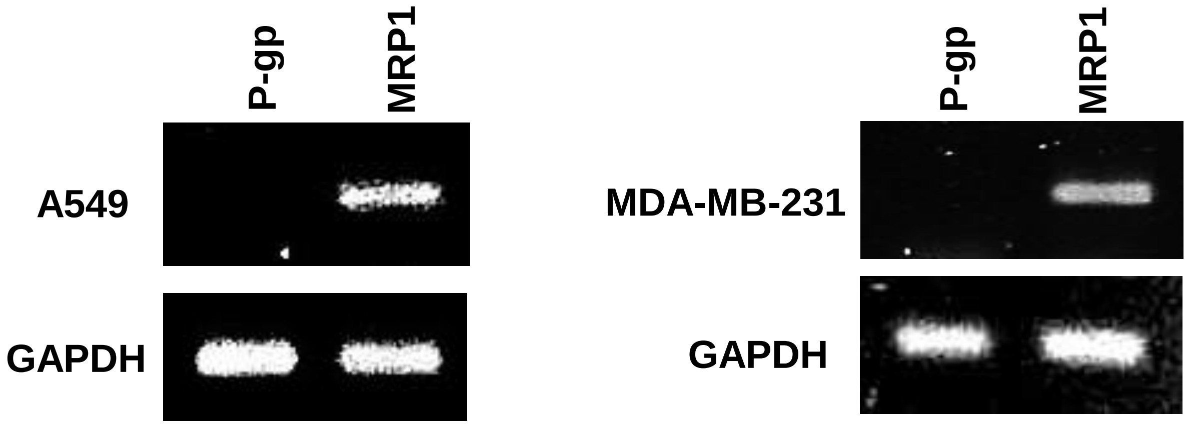

Figure S17. mRNA expression of efflux associated genes (*P-gp* and *mrp-1*) on cancer cells. *P-glycoprotein 1 (P-gp)* and *mrp-1* mRNA expression was compared by RT-PCR in cancer cells. GAPDH was used as a control. Only *mrp-1* mRNA expression was detectable in A549 and MDA-MB-231 cells.

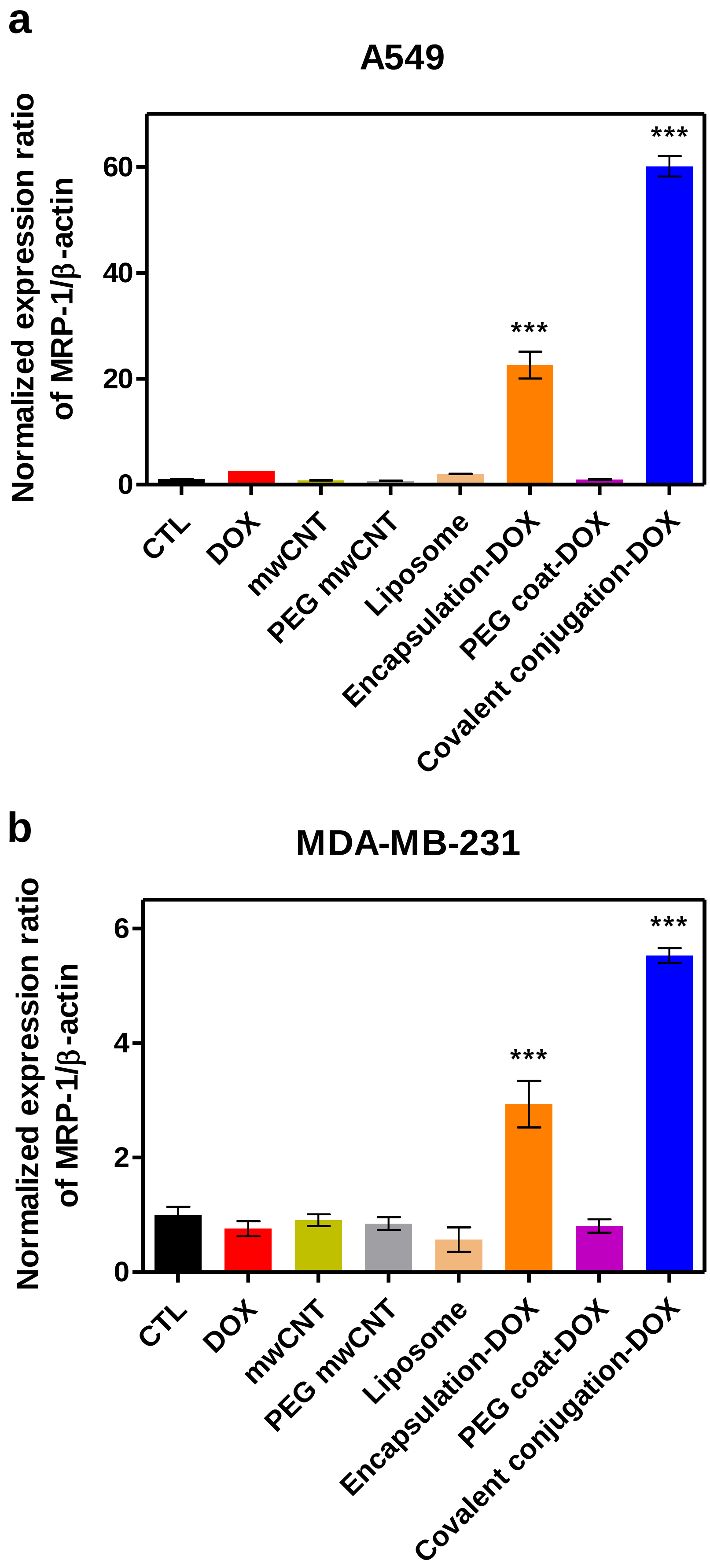

a
A549
Normalized expression ratio of MRP-1/β-actin
0
20
40
60
***
***
CTL
DOX
mwCNT
PEG mwCNT
Liposome
Encapsulation-DOX
PEG coat-DOX
Covalent conjugation-DOX
b
MDA-MB-231
Normalized expression ratio of MRP-1/β-actin
0
2
4
6
***
***
CTL
DOX
mwCNT
PEG mwCNT
Liposome
Encapsulation-DOX
PEG coat-DOX
Covalent conjugation-DOX

Figure S18. MRP-1 protein expression in cancer cells. (a and b) Densitometric measurement of MRP-1 protein expression in **(a)** A549 and **(b)** MDA-MB-231 cells. Changes in MRP-1 protein levels in A549 and MDA-MB-231 cells were analyzed (after 24 hrs) by western blot, and normalized with respect to β-actin. The relevant MRP-1 protein expression was calculated by the intensity of the bands. Data represent mean ± SEM (n=3).

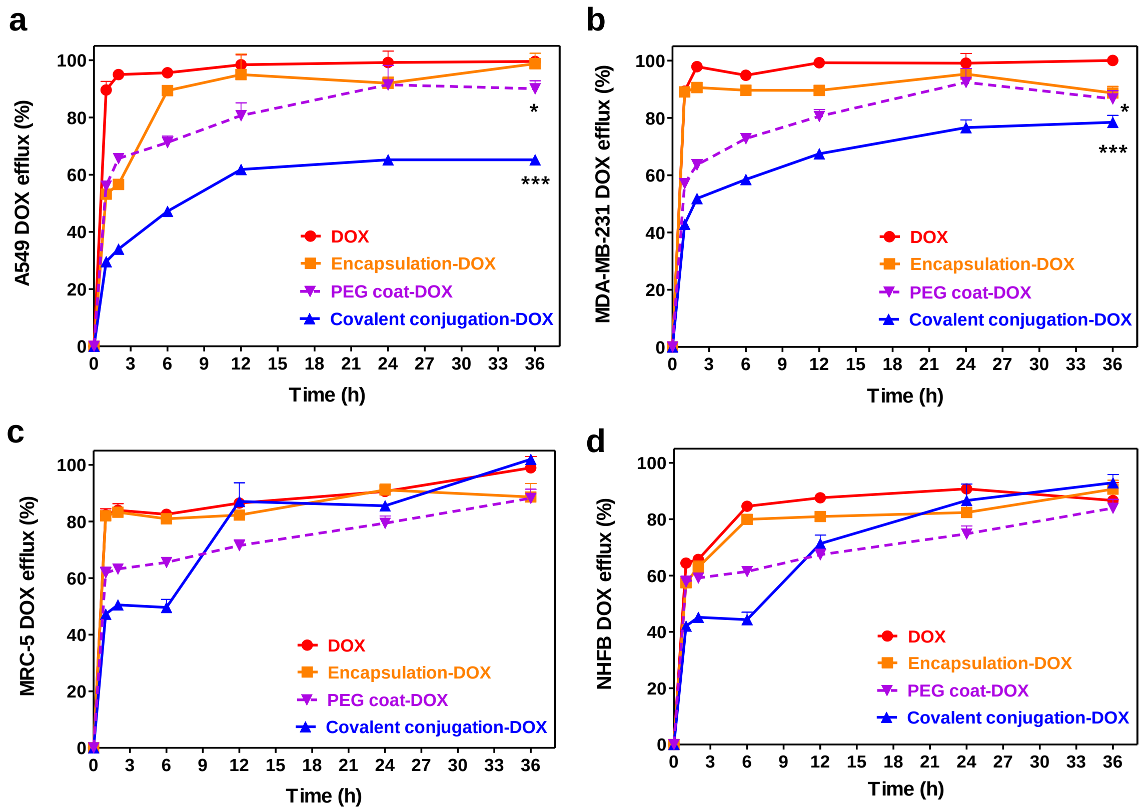

Figure S19. Extracellular pathway (drug clearance) of nanodrugs. Time dependent extracellular DOX clearance from cancer (**a**: A549, **b**: MDA-MB-231) and normal cells (**c**: MRC-5, **d**: NHFB). After 36 hrs, 25~40 % of the total DOX amount in nanodrug samples with covalent conjugation (blue line) still remained in the intracellular region, whereas free DOX (red) and encapsulation (orange) cleared relatively quickly from cells compared to PEG coat (purple), which showed 10 % of drugs left over. All data represent the mean SEM (n = 5). *, ** and *** correspond to $p < 0.05$, $p < 0.01$ and $p < 0.001$, respectively, as compared with free DOX (i.e., red).

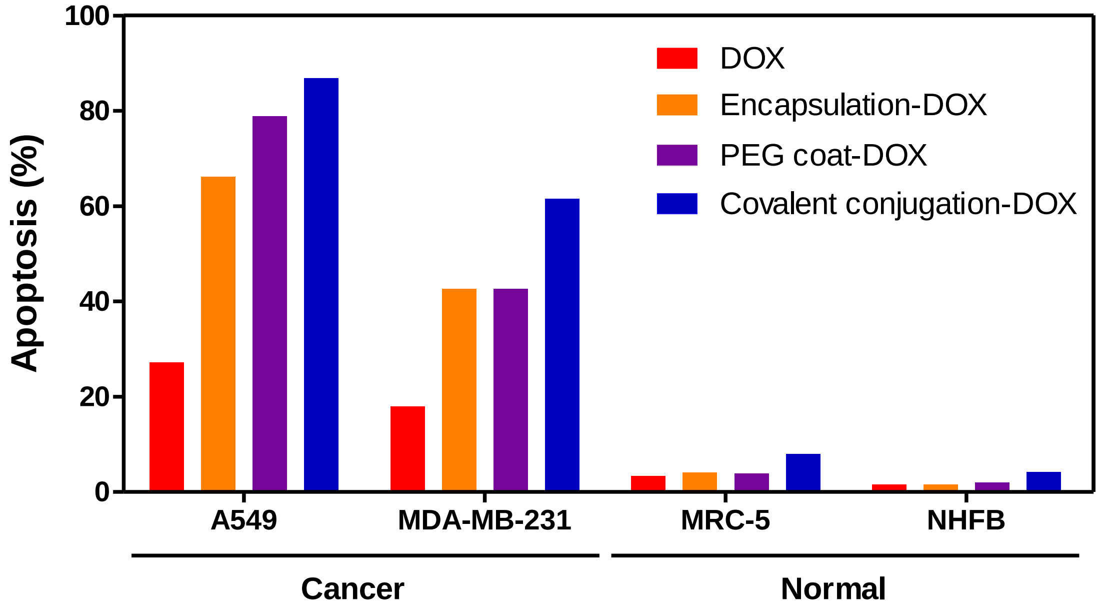

Figure S20. Percent increase in apoptosis in cancer cells compared to normal cells for the different types of nanodrugs (e.g., covalent conjugation, PEG coat and liposomal encapsulation). Specifically, covalent conjugation-DOX (blue marker) elicited the highest apoptosis in cancer cells, but yielded negligible apoptosis in normal cells.

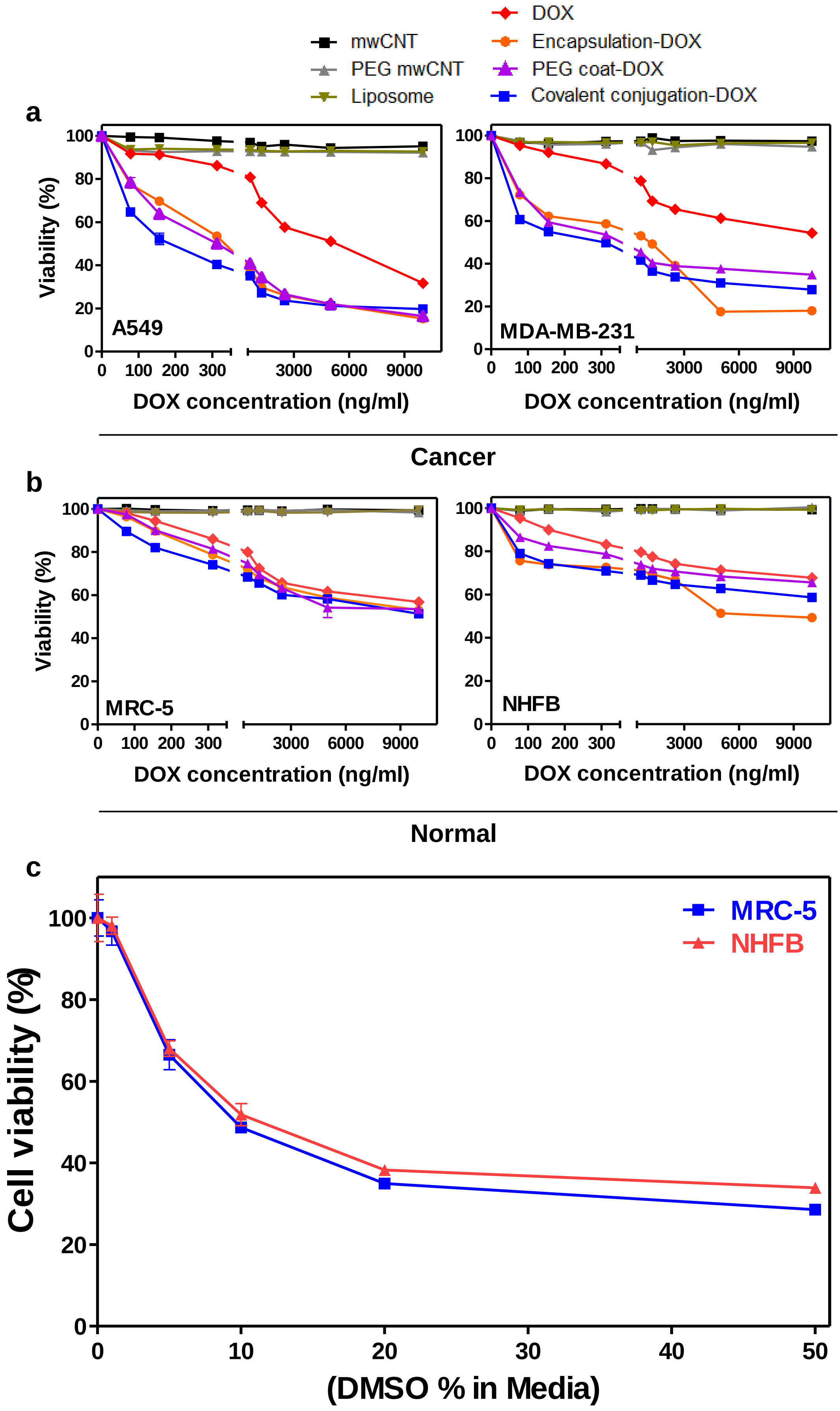
mwCNT
PEG mwCNT
Liposome
DOX
Encapsulation-DOX
PEG coat-DOX
Covalent conjugation-DOX
a
Viability (%)
A549
MDA-MB-231
DOX concentration (ng/ml)
Cancer
b
Viability (%)
MRC-5
NHFB
DOX concentration (ng/ml)
Normal
c
Cell viability (%)
MRC-5
NHFB
(DMSO % in Media)

Figure S21. Differential cytotoxicity on cancer and normal cells by nanodrugs. MTT results of **(a)** A549 and MDA-MB-231 cancer cells showed that covalent conjugation-DOX (blue) recorded the lowest cell density (i.e., increased cytotoxicity to cancer cells) after 48 hrs. **(b)** However, negligible difference of cytotoxicity was identified in normal cells (MRC-5 and NHFB) for the various types of nanodrugs (e.g., covalent conjugation, PEG coat conjugation and liposomal encapsulation). **(c)** DMSO toxicity for normal cells (MTT). Maximum % of cell viability after 24 hrs treatment with DMSO was 28~30% in MRC-5 and NHFB.

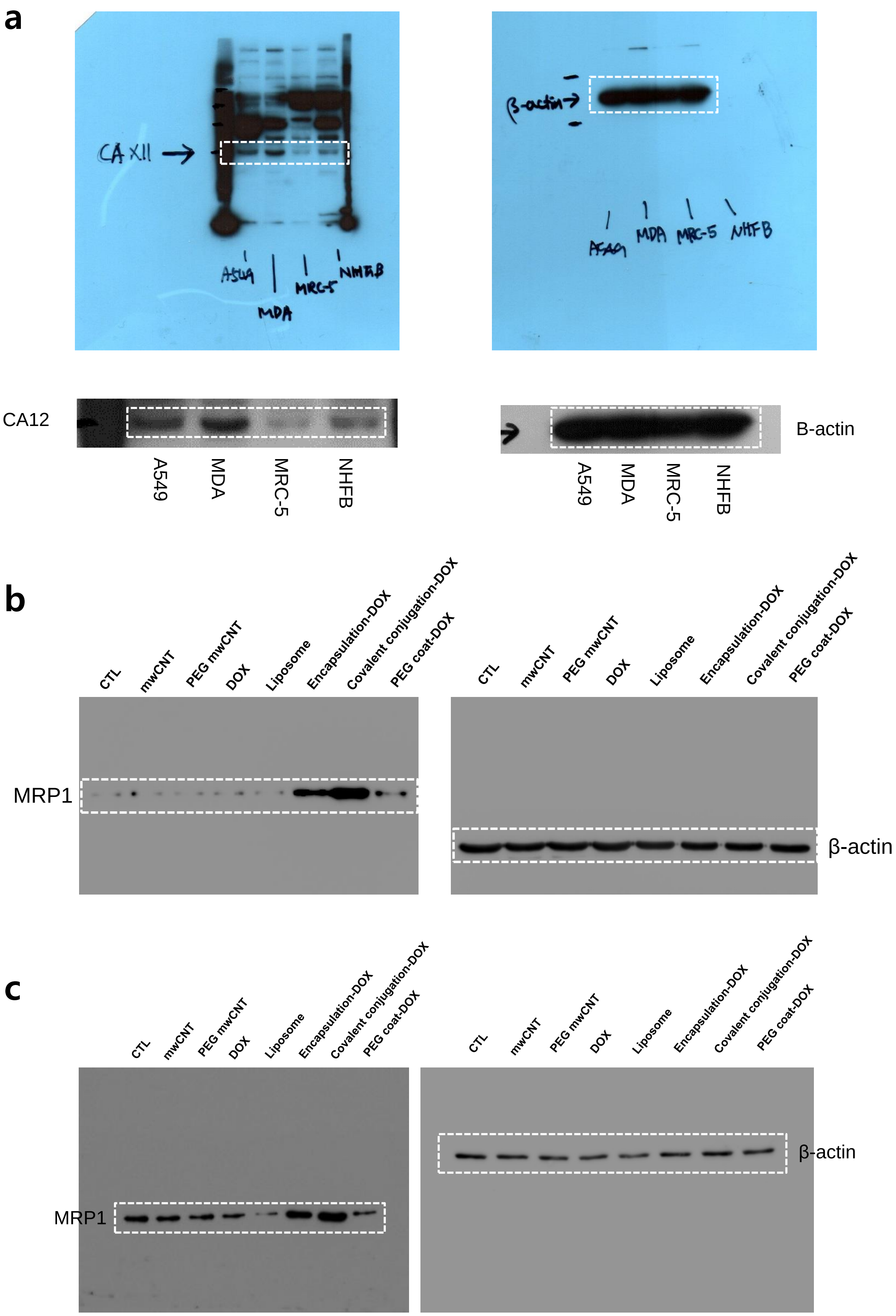
a
CA XII
β-actin
CA12
A549
MDA
MRC-5
NHFB
B-actin
b
CTL
mwCNT
PEG mwCNT
DOX
Liposome
Encapsulation-DOX
Covalent conjugation-DOX
PEG coat-DOX
MRP1
β-actin
c
MRP1
β-actin

Figure S22. According to the policy of *Scientific Reports* the full-length blots of the Western Blot analysis shown in **(a)** Fig. 2d, **(b)** Fig. 5c and **(c)** Fig. 5d are included in the Supplementary Information.
